# BACH1 deficiency improves placental angiogenesis via SLC25A51-mediated mitochondrial NAD^+^ transport in intrahepatic cholestasis of pregnancy

**DOI:** 10.1186/s10020-025-01215-4

**Published:** 2025-05-01

**Authors:** Shengpeng Li, Weiying Zhu, Zhixuan Xing, Dan Chen, Huimin Zhao, Yanli Zhang, Wenlong Zhang, Jiaojiao Sun, Yaxian Wu, Ling Ai, Qingfeng Pang

**Affiliations:** 1 Wuxi School of Medicine, Jiangnan University, 1800 Lihu Avenue, Wuxi, 214122 Jiangsu province PR China; 2 Medical Basic Research Innovation Center for Gut Microbiota and Chronic Diseases, Ministry of Education, Jiangnan University, 1800 Lihu Avenue, Wuxi, 214122 Jiangsu province PR China; 3 Department of Obstetrics, Maternity and Child Health Care Affiliated Hospital, Jiaxing University, NO.2468 East Central Road, South Lake District, Jiaxing, 314000 PR China

**Keywords:** BACH1, SLC25A51, NAD^+^, Angiogenesis, Intrahepatic cholestasis of pregnancy

## Abstract

**Background:**

Placental angiogenesis is particularly important in the treatment of intrahepatic cholestasis of pregnancy (ICP). Although BACH1 has been implicated in angiogenesis associated with cardiovascular diseases, its specific role and underlying mechanisms in ICP remain unclear. This study aims to investigate the role of BACH1 in ICP.

**Methods:**

The study used clinical samples and two distinct mouse models of ICP to validate BACH1 alterations in ICP through immunohistochemistry (IHC), immunofluorescence (IF), and western blot (WB) analyses. Subsequently, global BACH1-knockout mice were employed to investigate the phenotypic effects of BACH1 deficiency on ICP progression. The molecular mechanisms underlying the regulatory role of BACH1 in ICP were further elucidated using multi-omics approaches (e.g., transcriptomics and proteomics), combined with dual-luciferase reporter assays and electrophoretic mobility shift assays (EMSA).

**Results:**

The expression of BACH1 was significantly upregulated in ICP, and its expression level positively correlated with clinicopathological indicators of ICP. Experiments using BACH1-knockout mice demonstrated that BACH1 deletion effectively ameliorated ICP-related placental tissue damage and significantly enhanced the expression levels of angiogenesis markers such as vascular endothelial growth factor (VEGF). Mechanistic investigations indicated that BACH1 deficiency activated the transcriptional expression of solute carrier family 25 member 51 (SLC25A51), thereby promoting the mitochondrial transport of nicotinamide adenine dinucleotide (NAD^+^), restoring mitochondrial function, and improving the activities of electron transport chain complexes I, II, and IV. Notably, BACH1 deficiency promoted taurocholic acid (TCA)-induced proliferation of human umbilical vein endothelial cells (HUVECs), whereas this phenotype could be reversed by shRNA-mediated knockdown of SLC25A51. Further studies confirmed that administration of the specific BACH1 inhibitor HPPE effectively alleviated TCA-induced suppression of HUVECs proliferation.

**Conclusions:**

BACH1 may suppress placental angiogenesis by inhibiting the transcriptional expression of SLC25A51, making it a potential therapeutic target. Specifically, pharmacological inhibition of BACH1 could provide a targeted therapeutic strategy for placental angiogenesis associated with ICP.

**Graphical Abstract:**

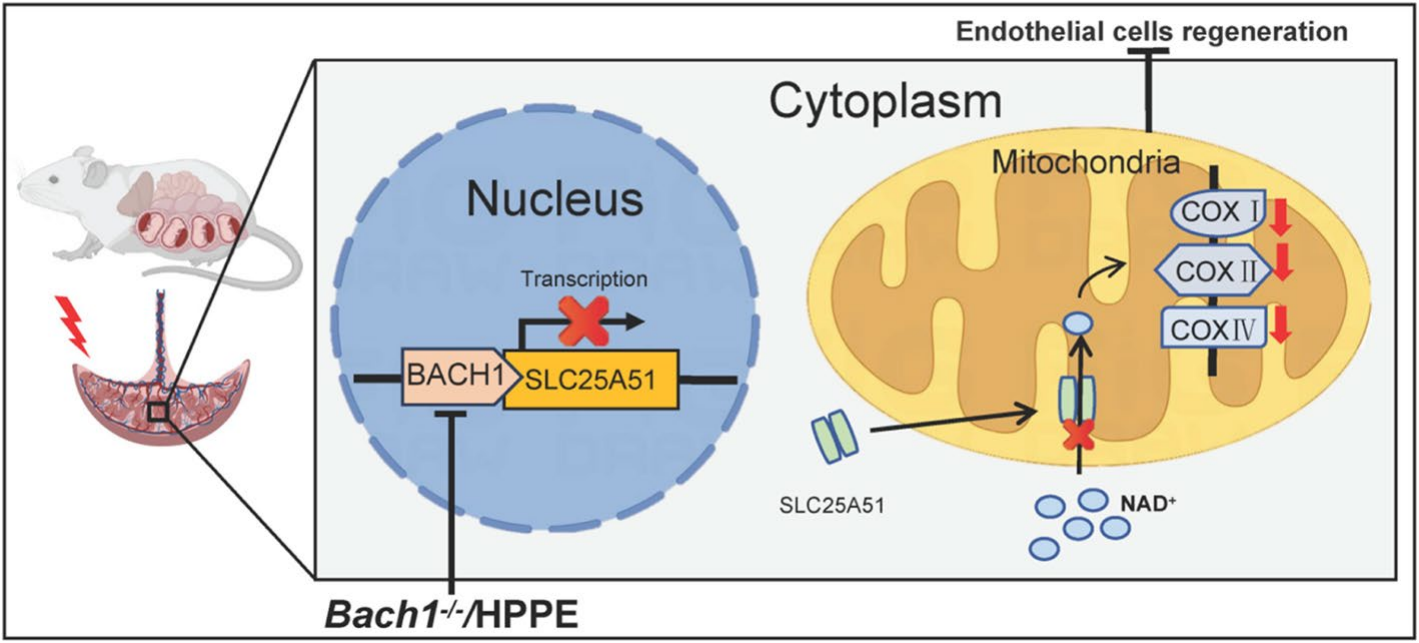

**Supplementary Information:**

The online version contains supplementary material available at 10.1186/s10020-025-01215-4.

## Introduction

Intrahepatic cholestasis of pregnancy (ICP) is an idiopathic liver condition that arises during gestation and is associated with adverse pregnancy outcomes, including fetal distress, premature delivery, stillbirth, and postpartum hemorrhage (Ovadia et al. 2016). Despite extensive research efforts, satisfactory clinical interventions for ICP remain elusive (Chappell et al. 2019).

Placental angiogenesis is not only a characteristic of ICP but also a significant contributor to adverse pregnancy outcomes. A normal placenta exhibits a well-organized vascular architecture comprising large blood vessels. On the contrary, the placental vascular density in each villus gradually decreased in patients with mild to severe ICP. This deficiency may lead to placental fetal perfusion damage, fetal hypoxia and sudden intrauterine death (Du et al. 2014; Wang et al. 2021a, b). Emerging evidence suggests that placental vascular endothelial cells (PVECs) may play a central role in the pathophysiology of placental insufficiency associated with ICP (Han et al. 2018). They require extensive transport of various substances, necessitating a high demand for ATP (Yang et al. 2002a, b). Therefore, PVECs, which are rich in mitochondria, exhibit mitochondrial damage including respiratory function impairment associated with placental angiogenesis in ICP (Wu et al. 2016).

Nicotinamide adenine dinucleotide (NAD^+^) serves as an essential coenzyme that orchestrates redox homeostasis and metabolic signaling pathways (Kory et al. 2020), while simultaneously functioning as a central regulator of cellular bioenergetics, mitochondrial function, and epigenetic modulation (Nasuhidehnavi et al. 2025). The latest research shows that a decrease in mitochondrial NAD^+^ content impairs mitochondrial respiratory function (Bhatti et al. 2017), resulting in reduced ATP levels and elevated ROS production (Song et al. 2021). Conversely, augmenting mitochondrial NAD^+^ levels enhances mitochondrial respiration (Tannous et al. 2024) and mitigates ROS generation (Hu et al. 2022). Solute carrier family 25 member 51 (SLC25 A51) resides on the inner mitochondrial membrane, primarily as an NAD^+^ transporter in mammalian mitochondria. This establishes the feasibility of direct NAD^+^ exchange between the mammalian cytoplasm and mitochondria. Additionally, SLC25 A51 is functionally associated with the tricarboxylic acid cycle and mitochondrial respiratory function (Luongo et al. 2020; Li et al. 2023). Loss of SLC25 A51 impairs mitochondrial biogenesis (Güldenpfennig et al. 2023). However, the underlying mechanism of SLC25 A51 deficiency in ICP has not been defined.

BTB and CNC homology 1 (BACH1) is a transcriptional repressor of the Cap‘n’collar (CNC) family. BACH1 enhances oxidative stress and regulates angiogenesis (Zhang et al. 2018, He et al. 2023). BACH1 is also considered to potentially regulate mitochondrial respiratory function. Specifically, BACH1 knockout reverses Pb-induced suppression of mitochondrial electron transport chain (ETC) transcription and expression, increasing ATP levels (Li et al. 2022). Additionally, downregulating BACH1 alleviates mitochondrial dysfunction and synaptic impairment induced by oxidative stress (Du et al. 2022). However, the role of BACH1 in mitochondrial respiratory function in the placental vasculature remains unclear.

In this study, we aim to investigate the role of BACH1 in ICP. Our data demonstrated that BACH1 is significantly elevated in placental vascular endothelial cells during ICP. As a nuclear transcriptional repressor, BACH1 directly inhibits the transcription of SLC25 A51, reducing NAD^+^ content in the mitochondria and suppressing the expression of genes associated with the mitochondrial respiratory chain. This study confirms that inhibiting BACH1 promotes placental angiogenesis and provides a theoretical basis for clinical intervention.

## Method Details

### Experimental model and study participant details

Placental samples were obtained from patients with ICP (*n* = 33) and healthy donors (normal; *n* = 36) at Jiaxing Maternal and Child Health Hospital (Table S1). Written informed consent was obtained from all participants. All related experiments were approved by the Ethics Committee of Jiaxing Maternal and Child Health Hospital, Jiaxing, China (ethics review no. 2023-036) and adhered to the principles outlined in the Declaration of Helsinki of the World Medical Association. ICP was defined according to the guidelines for the clinical diagnosis, treatment, and management of ICP (2024) (Hobson et al. 2024).

The animal experiments were approved by the Ethics Committee of Jiangnan University (approval no. JN. No 20211215 m0901001) and conducted following established protocols. Pathogen-free mice (6–8 weeks old) with a C57BL/6 background, including WT and Bach1-knockout (Bach1^-/-^) mice (Sun et al. 2025), were obtained from the Model Animal Research Center, MARC, Nanjing (project no. FW-20181225-01 TY).

Human umbilical vein endothelial cells (HUVECs) and human embryonic kidney 293 T cells (HEK-293 T) (ATCC, Manassas, VA, USA) were maintained in Dulbecco’s modified Eagle’s medium (DMEM; Gibco, Grand Island, NY, USA) supplemented with 10% fetal bovine serum (Yeasen Biotech Co. Ltd., Shanghai, China) and 1% penicillin/streptomycin (Gibco). The cells were incubated in a cell culture chamber at 37 °C with 5% CO_2_. HUVECs were plated in 12-well plates at a density of 1 × 10^5^ cells/mL. HUVECs were pretreated with HPPE (5 µM) for 4 h and stimulated with taurocholic acid (TCA) (100 µM) for 24 h.

### Enzyme-linked immunosorbent assay (ELISA)

BACH1 protein abundance in placentas was detected using ELISA kits (Meimian, Yancheng, China, MM-51647H1) following the manufacturer’s protocol. Absorbance was measured at 450 nm using a Gen5 plate reader. The background values at 570 nm were subtracted from those at 450 nm.

### Proteomic analysis

Five healthy pregnant women and five patients with ICP were recruited at Jiaxing Maternal and Child Health Hospital. Placental samples were collected and transferred to liquid nitrogen within 3 min of delivery. Reverse-phase chromatography and mass spectrometry were conducted by Shanghai Luming Biological Technology. ProteomeDiscoverer version 2.4.1.15 (ThermoFisher Scientific) was used to search all raw data against the UniProt Mus musculus database, with trypsin digestion specificity and cysteine alkylation as fixed modifications. Tandem mass tags were selected for protein quantification. A global false discovery rate (FDR) of 0.01 was applied, and protein groups required at least one peptide for quantification. Gene Ontology (GO) and Kyoto Encyclopedia of Genes and Genomes (KEGG) pathway analyses were performed to categorize the significantly differentially abundant proteins.

### RNA-seq

The keywords “intrahepatic cholestasis of pregnancy (ICP)” was searched on the National Center for Biotechnology Information (NCBI) public data platform GEO (Gene Expression Omnibus), with “*Homo sapiens*” selected as the species. The gene chip data GSE46157 were downloaded (https://www.ncbi.nlm.nih.gov/geo/geo2r/?acc=GSE46157). GO and KEGG analyses were performed using the DAVID online software. GO analysis software was used to analyze the biological processes, molecular functions, and cellular components of the differentially abundant genes. *p* < 0.05 was considered statistically significant (Feng et al. 2022).

### Vascular separation of human placenta

Human placental tissues were maintained in ice-cold physiological saline prior to processing. Under stereomicroscopic guidance, vascular branching structures were meticulously dissected using microsurgical instruments. The isolated blood vessel specimens were promptly dissected free of adherent connective tissue using atraumatic techniques, followed by cryopreservation at −80°C in validated storage conditions until experimental utilization (Zhang et al. 2024).

### Mouse models

One week before mating, female C57BL mice were fed a specified diet, either a standard diet (SD) or 0.5% CA-supplemented SD (CA, Wuxi Fanbo Biotechnology Co., Ltd); this diet was continued for 25 days. WT mice were randomly divided into two groups (*n*=6/group): WT+SD and WT+CA. Bach1^-/-^ mice were randomly divided into two groups (*n*=6/group): Bach1^-/-^+SD and Bach1^-/-^+CA (Pataia et al. 2020).

Adult mice were caged at a 2:1 male-to-female ratio, and vaginal plugs of female mice were examined the following morning as an indicator of successful conception. Beginning on GD 12.5, estradiol (E2; Sigma, St. Louis, MO, USA; Catalog No. 1260001-150 mg, 5 mg/kg) dissolved in propylene glycol (PG; final concentration: 1.0 mg/mL) was subcutaneously injected into WT mice. Mice were randomly divided into two groups (*n* = 6/group): a PG control group and an E2-treated group (Wang et al. 2016).

At 17.5 and 18.5 days of gestation, pregnant mice were anesthetized with isoflurane, and blood samples were collected via retro-orbital bleeding. Following cervical dislocation, maternal liver tissues, fetuses, and placental samples were harvested. Placental specimens were weighed, and their diameters were measured. Fetuses were individually weighed, and crown-rump lengths were recorded.

### Histological analysis

Murine placental and liver tissues were fixed in 4% paraformaldehyde, dehydrated, embedded in paraffin, and cut into sections (4 µm) for hematoxylin and eosin staining.

### Immunohistochemistry staining

Placental tissue sections were deparaffinized, rehydrated, and subjected to antigen retrieval in 10 mM sodium citrate buffer heated to 95 °C for 30 min. Non-specific binding was blocked using normal goat serum for 20 min at 37 °C. The primary antibody targeting BACH1 (#80593-1-RR, diluted 1:200; Proteintech, Wuhan, China) was incubated overnight, followed by incubation with horseradish peroxidase-labeled secondary antibody at room temperature for 1 h. Subsequently, the sections were stained with 3,3’-diaminobenzidine (DAB; Nanjing Jiancheng, Nanjing, China) for 5 min and counterstained with hematoxylin.

### Biochemical index detection

Alanine aminotransferase (ALT), aspartate aminotransferase (AST), and total bile acids (TBA) levels in serum and liver tissue were measured using commercial test kits (Nanjing Jiancheng Bioengineering Institute, Nanjing, China) according to the following protocols: ALT/GPT was assessed using the microplate method (C009-2-1), AST/GOT was assessed using the microplate method (C010-2-1), and TBA (E003-2-1) was analyzed with the microplate method.

### Plasmid construction

The SLC25 A51 OE plasmid pcDNA3.1(+)-SLC25 A51-3×FLAG-P2 A-EGFP (forward primer [CMV-F], CGCAAATGGGCGGTAGGCGTG; reverse primer [EGFP-SEQR], GACACGCTGAACTTGTGGC) and the control vector H2713 pcDNA3.1(+)−3×FLAG-P2 A-EGFP were designed and constructed by ObiO Technology. HUVECs were placed in a six-well plate (2.5 × 10^5^ cells/well); 2 µg of DNA and 10 µL of transfection reagent (no. AD600150; Zeta Life) were added, and the plate was maintained at room temperature for 25 min according to the manufacturer’s protocol.

### Small interfering RNA (siRNA) transfection

HUVECs were transfected with BACH1- and SLC25 A51-targeting siRNAs (KeyGEN BioTECH, Nanjing, China) using a Zeta Life transfection reagent (#AD600150) according to established protocols. The transfected cells were maintained for 48 h before subsequent analysis. siRNA sequences targeting human BACH1 and SLC25 A51 are detailed in Supplementary Table S2.

### Gene knockdown methodology

Human umbilical vein endothelial cells (HUVECs) were transduced with lentiviral particles containing SLC25 A51-targeting short-hairpin RNA (shRNA) following manufacturer protocols. Stable clones were selected through puromycin resistance (2 µg/mL; Sigma-Aldrich, P8833) over 7–10 days. Successful knockdown was verified by Western blot analysis using anti-SLC25 A51 antibodies (1:1000 dilution), with β-actin serving as a loading control (Figure S8 A). The specific shRNA target sequences are detailed in Table S3.

### Mitochondrial isolation

Placental tissue and HUVECs were processed into cell suspensions using a tissue and cell mitochondrial kit (C3601, C3606; Beyotime Biotechnology, Shanghai, China). The suspensions were then centrifuged to remove the supernatant. A concentration of 1 mM phenylmethylsulfonyl fluoride (PMSF; 1 mM) was added to the suspensions. Subsequently, a pre-cooled mitochondrial separation reagent was introduced and homogenized in an ice bath for ten cycles. The resultant supernatant was transferred to a fresh centrifuge tube and centrifuged at 600 g for 5 min at 4 °C. The supernatant was centrifuged at 11,000 g for 10 min at 4 °C. The precipitate obtained contained isolated mitochondria.

### Mitochondrial NAD^+^ uptake assay

Isolated mitochondria were resuspended (50–200 µg) in MiR05 (60101-01, NextGen-O2k) containing 5 mM malate (T2S0850, TargetMol), 10 mM pyruvate (T4804, TargetMol), and 1 mM NAD^+^ (HY-B0445, MCE). The reaction mixture was agitated at 900 rpm, and the tube was briefly opened every 10 min to allow re-oxygenation. Mitochondria were granulated by centrifugation (14,000 g, 2 min), and NAD^+^ content in the solution was measured according to the manufacturer’s instructions (A114-1-1, Nanjing Jiancheng Bioengineering Institute).

### Mitochondrial ATP, SOD and ROS levels

Mitochondrial ATP, SOD, and ROS levels were quantified using respective Enhanced Assay Kits (Jiancheng Bioengineering, Nanjing) following the manufacturer’s protocols.

### Detection of cytosolic ROS in HUVECs

HUVECs were seeded in a 12-well plate (2.2 × 10^5^ cells/well). Upon reaching ~80–90% confluence, specific chemicals were added. After 24 h, the cells were incubated with diacetyldichlorofluorescein (DCFH-DA; 1:500, E004-1-1, Nanjing Jiancheng Bioengineering Institute) for 25 min at 37 °C in the dark. After a loading period of 30 min, the unincorporated dye was removed by washing with PBS. The cells were observed and photographed under a fluorescence microscope (Nikon, Japan).

### Detection of mitochondrial membrane potential

Mitochondrial membrane potential (MMP) was assessed using JC-1 dye, which selectively enters mitochondria and changes from red to green upon decreased MMP. Cells were seeded at 2.2 × 10^5^ cells/well in a 12-well plate; upon reaching 80–90% confluency, specific chemicals were added. After 24 h, the cells were incubated with JC-1 for 30 min at 37 °C in the dark. Next, the unincorporated dye was removed by washing with PBS. Finally, images were acquired and analyzed using a Zeiss fluorescence microscope (Axio Imager Z2).

### Measurement of the oxygen consumption rate (OCR)

The OCR was measured using an Oxygen Consumption Rate kit (600800, Cayman Chemical). Cells were seeded in a 96-well flat-bottom black microplate and incubated for 2 d. After adding the phosphorescent oxygen probe to the wells, 100 µL of mineral oil was layered on top of the medium in each well. The plate was read on an EPOCH2 microplate reader (BioTek) in kinetic mode at 37 °C for 2 h (one read/3 min, Ex 380/Em 650). Basal OCR levels of the cells were calculated from the slope of the kinetic curve, following the manufacturer’s instructions (Teng et al. 2024).

### 5-Ethynyl-2′-deoxyuridine (EdU) assay

HUVECs were treated with 10 μM EdU for 24 h, and 4% paraformaldehyde-fixed cells were permeabilized with 0.3% Triton X-100 for 15 min. Endogenous peroxidase activity was blocked before analysis. EdU detection was performed using the BeyoClick™ EdU Cell Proliferation Kit with DAB (Beyotime Biotechnology), according to the manufacturer’s instructions. The number of EdU+ cells was counted using Image-Pro Plus 6.0 (Media Cybernetics).

### Migration assay

In the wound healing assay, HUVECs (2×10^5^ cells/mL) were plated into six-well plates and cultured for 24 h until they reached 80–90% confluence. Subsequently, a wound was induced in the central area by scratching the monolayer with the tip of a standard 1-mL pipette. After washing away the cellular debris with PBS, fresh media was added. Images of the wound healing process were captured at 0 and 24 h using an inverted microscope (Axio Vert. A1; Zeiss, Oberkochen, Germany). The distances between the migrated cells were photographed and measured.

### Cell viability assay

HUVECs were seeded in 96-well plates (1 × 10^5^/mL) and pretreated with different concentrations of HPPE (1, 2.5, 5, 10, and 20 μM) for 4 h. Subsequently, TCA (100 μM) was administered for 24 h before adding 10 μL of CCK8 to each well and incubating at 37 °C for 2 h. Finally, the absorbance at 450 nm was measured using a UV-VIS spectrophotometer.

### Immunofluorescence staining

The sections and cells were stained with antibodies specific for CD31 (Abcam, ab76533, 1:200, USA), SLC25 A51 (Cusabio, CSB-PA875649LA01HU, 1:200, China), and BACH1 (Proteintech, 80593-1-RR, 1:200; Wuhan, China) overnight at 4 °C. After washing with PBS, sections and cells were incubated with fluorophore-conjugated secondary antibodies at room temperature. Nuclei were stained with DAPI for 5 min at room temperature. Finally, images were acquired and analyzed using a Zeiss fluorescence microscope (Axio Imager Z2).

### Western blot assay

Total proteins from placental tissue and cultured cells were extracted using radioimmunoprecipitation assay (RIPA) lysis buffer (Vazyme Biotech Co., Ltd., China, Nanjing). Protein samples (20 µg) were fractionated using 10% SDS–PAGE and transferred to nitrocellulose membranes. Subsequently, the primary antibodies and corresponding secondary antibodies were added. The primary antibodies used are listed in Table S4.

### Reverse transcription quantitative polymerase chain reaction (RT-qPCR)

Placental blood vessels from pregnant women, placental tissue from mice, and HUVECs were sonicated in TRIzol reagent (R411-01, Vazyme Biotech Co., Ltd.) and homogenized according to the manufacturer’s protocol. RT-qPCR was performed using the PrimeScriptRT kit (R101-02, Vazyme Biotech Co., Ltd.), LightCycler 480 II real-time system (Roche Diagnostics, Shanghai, China), and SYBR mix (11201ES03; Yeasen Biotech Co. Ltd.). The comparative cycle threshold method was used to determine the relative mRNA expression levels, with GAPDH serving as the internal standard. The primer sequences are listed in Table S5.

### Luciferase assays

The JASPAR database (http://jaspar.genereg.net/) was used to predict the binding sites of BACH1 in the *SLC25 A51* promoter. The 2 kb WT and mutant SLC25 A51 promoters were cloned into the pGL3-basic luciferase reporter vector (Promega Corporation, USA). The recombinant constructs were co-transfected with the pRL-TK plasmid expressing Renilla luciferase (Promega Corporation) or BACH1-OE plasmid into HEK-293 T cells. Firefly and Renilla luciferase activities were measured 24 h after transfection using a Dual-Luciferase Reporter Gene Assay Kit (11402ES60; Yeasen Biotech Co. Ltd.).

### Protein purification

Translate the plasmid containing Bach1 into *Escherichia coli*. *Escherichia coli* was cultured in LB medium until OD600 reached 0.6–0.8. Subsequently, induce BACH1 expression using IPTG (500 μM). Then lyse the cells to release the proteins. Finally, purify BACH1 using methods such as chromatography, filtration, and concentration.

### EMSA

Based on bioinformatic predictions of the binding sites for BACH1 and SLC25 A51, WT and mutant probe sequences were synthesized by GENEWIZ. Equal concentrations (10 μM) of the two complementary oligonucleotides were mixed in annealing buffer for DNA oligonucleotides (5×) (Beyotime Biotechnology) to generate the corresponding double-stranded DNA probes. EMSA was performed (Beyotime Biotechnology). Briefly, DNA probes were radiolabeled with [γ−32P] ATP and resolved on a 6% native polyacrylamide gel following pre-electrophoresis in 0.5× TBE buffer for 1 h. Subsequently, after staining with ethidium bromide for 30 min, the gels were subjected to UV cross-linking under an automatic image analysis system, and photographs were acquired (He et al. 2024a, b).

### Quantification and statistical analysis

Data were expressed as mean ± standard deviation. The results were compared using a two-tailed Student’s *t-*test (for two groups) or one-way analysis of variance (ANOVA), followed by Tukey’s post-hoc analysis (for more than two groups). Pearson correlation coefficients were calculated for the correlation matrices. Statistical significance was set at *p < 0.05*. Prism version 9.0 (GraphPad, La Jolla, CA, USA) was used for data analysis and image processing.

## Results

### BACH1 level is elevated in the placental tissue of patients with ICP and in mouse models

**Fig. 1:**
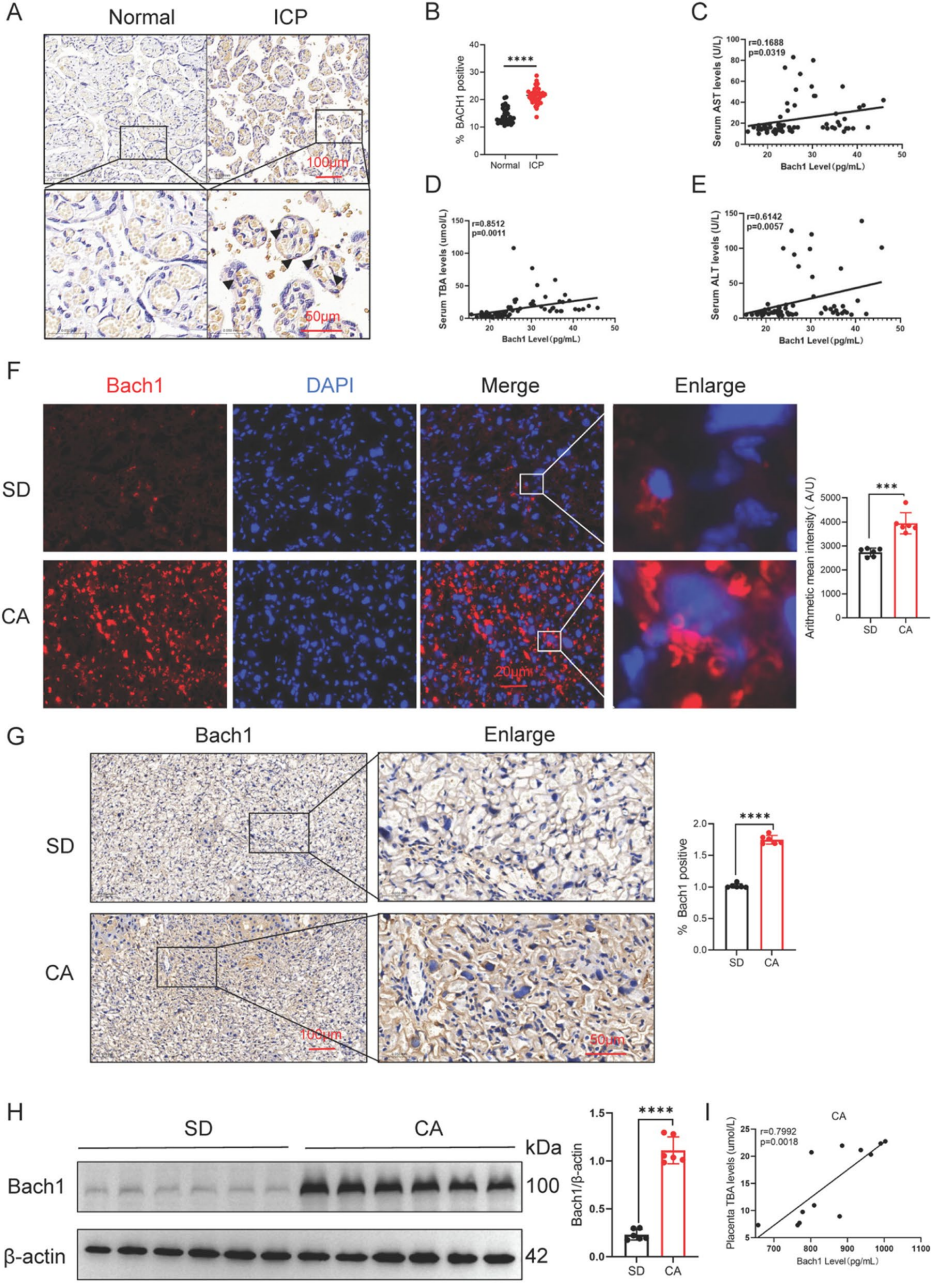
BACH1 expression is elevated in both human and mouse placental tissues with ICP. **A**, **B** Immunohistochemistry of BACH in 136 normal pregnant women and 33 patients with ICP; scale bar: 100 μM, 50 μM. **C**–**E** Positive correlation between BACH1 protein abundance and serum TBA, ALT, or (AST content in placenta of 36 normal pregnant women and 33 patients with ICP; Spearman correlation analysis. **F** BACH1 (red) immunostaining in placentas of WT mice treated with CA; DAPI (blue); scale bar: 20 μM; *n* = 6. **G** BACH1 protein abundance in placentas of WT mice treated with CA; scale bar: 100 μM, 50 μM; *n* = 6. **H** Western blotting for BACH1 in placentas of WT mice treated with CA (*n* = 6). **I** Strong positive correlation between BACH1 and TBA content in placenta of WT mice treated with CA; Spearman correlation. Unpaired two-tailed Student’s *t*-test or Spearman correlation analysis; ***p < 0.001, ****p < 0.0001

This study employed immunohistochemical techniques to analyze placental tissues from 36 cases in the healthy control group and 33 cases in the ICP group. The results demonstrated that the content of BACH1 protein in placental tissues of the ICP group was significantly elevated compared to the healthy control group (Fig. 1A, B). Further analysis revealed significant positive correlations between BACH1 protein expression and TBA (*r*=0.8512, *p*=0.0011), ALT (*r*=0.6142, *p*=0.0057), and AST (*r*=0.1688, *p*=0.0319) levels (Fig. 1C-E).

**Fig. 2:**
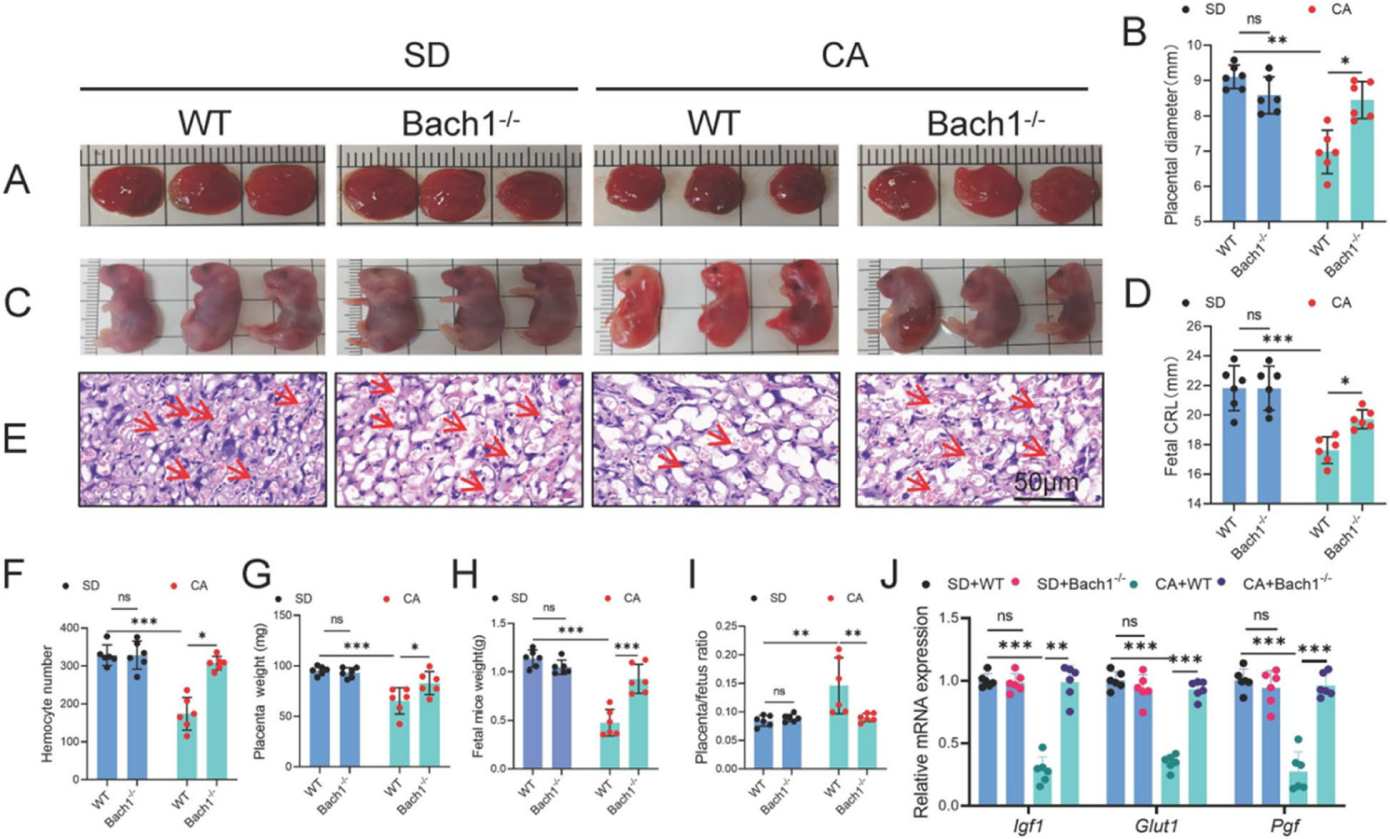
Bach1 knockout improves placental function in CA-induced mice. **A**, **B** Placental diameter on GD18.5 (*n* = 6). **C**, **D** Crown–rump length of fetal mice on GD18.5 (*n* = 6). **E**, **F** The hemocyte number in the placenta of C57BL/6 mice that were either WT or Bach1^-/-^ and treated with CA (*n* = 6) was analyzed using ImageJ software (Scale bar: 50 μM). **G**–**I** Fetal mouse weight, placental weight, and placenta/fetus ratio (*n* = 6). **J** *Igf1*, *Glut1,* and *Pgf* mRNA expression of placenta (*n* = 6). One-way analysis of variance (ANOVA) followed by Tukey’s post-hoc analysis; ns: not significant, *p < 0.05, **p < 0.01, ***p < 0.0001

We established two distinct mouse models of ICP through dietary CA supplementation and intraperitoneal E2 injection, respectively (Figure S1 A-H and S1I-P). Analysis of CA-treated placental tissues demonstrated: (1) Consistently elevated content of BACH1 protein levels across immunofluorescence, immunohistochemical, and WB analyses compared to the SD group (Fig. 1F); (2) A significant positive correlation between placental BACH1 expression and serum TBA concentrations (Fig. 1I). Remarkably, this association was independently validated in the E2-induced ICP model (Figure S2). Collectively, these findings establish that BACH1 is significantly upregulated in ICP placental tissues.

### Bach1 knockout attenuates CA-induced placenta damage in mice

To elucidate the role of BACH1 in ICP, this study employed CA induction to establish a BACH1^-/-^ ICP mouse model (Figure S3). Compared with the WT+CA group, the BACH1^-/-^+CA group exhibited significantly increased placental diameter (Fig. 2A, B) and enhanced fetal crown-rump length (Fig. 2C, D). Further analysis using ImageJ software in histology revealed a marked elevation in placental hemocyte number in this group compared to the WT+CA group. (Fig. 2E, F). Under CA treatment conditions, Bach1^-/-^ significantly increased placental weight (Fig. 2G) and fetal weight (Fig. 2H), while showing a decreasing trend in fetal-placental weight ratio (Fig. 2I). Bach1^-/-^ significantly upregulated mRNA expression levels of growth factor Igf1, glucose transporter Glut1, and placental growth factor Pgf in CA-treated placental tissues (Fig. 2J). These results collectively indicate that Bach1 deficiency effectively ameliorates CA-induced placental damage.

### Bach1 knockout promotes CA-induced placental angiogenesis

**Fig. 3:**
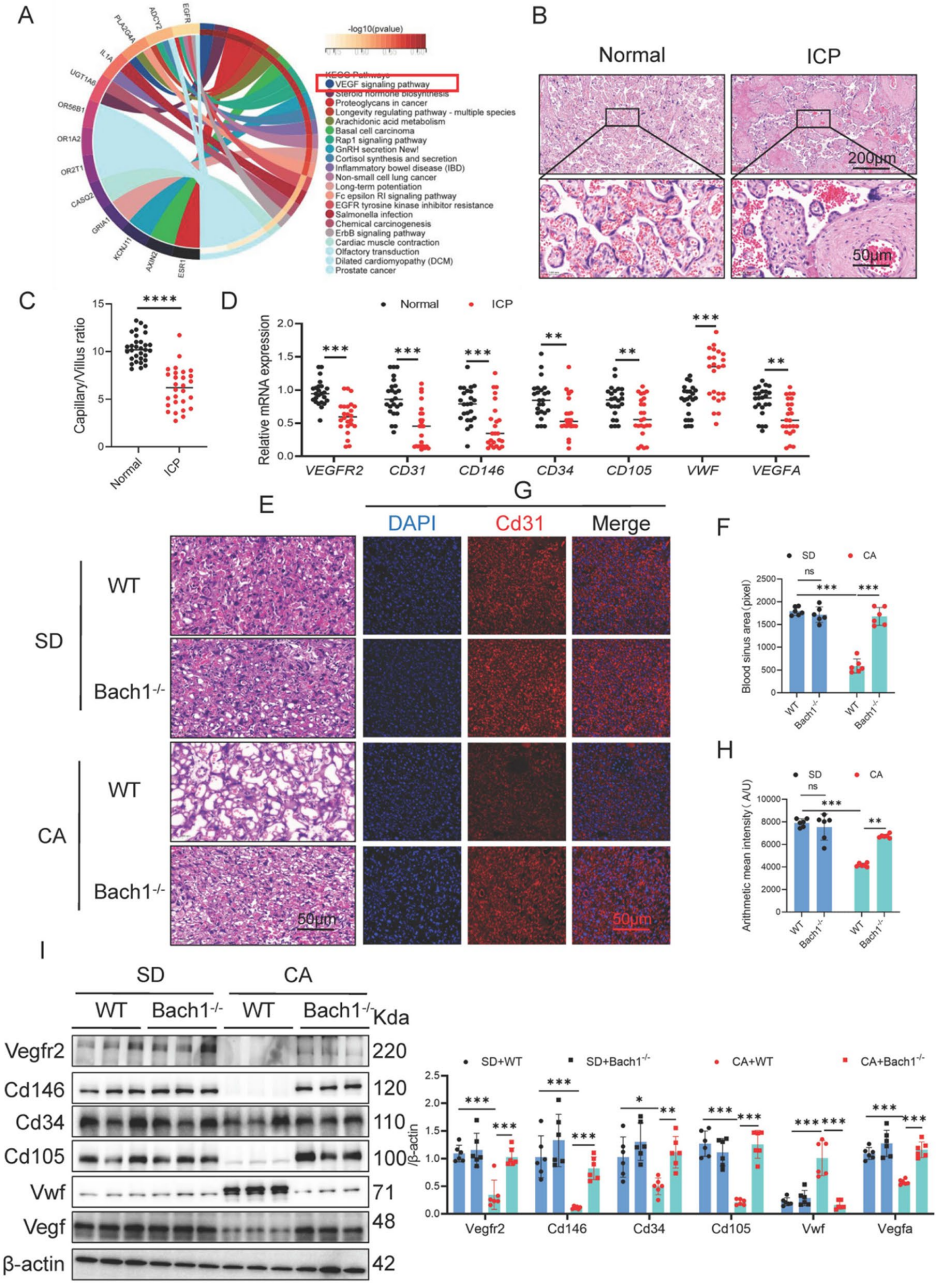
Bach1 knockout promotes expression of placental angiogenesis-related genes in CA-induced mice. **A** Enrichment of DEGs between placentas of normal pregnant women and patients with ICP; data from GSE46157. **B**, **C** H&E staining of blood vessels in the placenta of 36 normal pregnant women and 33 patients with ICP (scale bar: 200 μM;50 μM). **D** *VEGFR2*, *CD31*, *CD146*, *CD34*, *CD105*, *vWF*, and *VEGFA* mRNA expression in the placental vessels of patients with ICP (*n* = 24). **E**, **F** Blood sinus area in placenta of wild-type (WT) or Bach1^-/-^ C57BL/6 mice treated with CA (*n* = 6). **G**, **H** CD31 (red) immunostaining in placenta of WT or Bach1^-/-^ C57BL/6 mice treated with CA; DAPI (blue); scale bar: 50 μM; *n* = 24. **I** Western blotting results for VEGFR2, CD146, CD34, CD105, vWF, and VEGFA in placenta of WT or Bach1^-/-^ C57BL/6 mice treated with CA (*n* = 6). Unpaired two-tailed Student’s *t*-test or one-way analysis of variance (ANOVA) followed by Tukey’s post-hoc analysis; ns: not significant, *p < 0.05, **p < 0.01, ***p < 0.0001

Transcriptome data from placental tissues of patients with ICP (GSE46157) were analyzed. KEGG pathway analysis showed that VEGF signaling pathway was enriched (Fig. 3A). The capillary-to-villus ratio in the placenta was significantly decreased (Fig. 3B, C). In the vascular tissues of the ICP group, significant downregulation of VEGFA and VEGFR2 mRNA expression was observed, along with markedly reduced mRNA expression of endothelial cell markers (CD31, CD34, CD105, and CD146) (Fig. 3D). Notably, the mRNA expression of the endothelial cell-secreted factor vWF was significantly elevated in the vascular tissues of the ICP group (Fig. 3D).

The H&E staining results demonstrated that the Blood sinus area in Bach1^-/-^+CA group was significantly increased compared to the WT+CA group (Fig. 3E, F). Molecular analysis revealed that mRNA expression levels of VEGFA and VEGFR2 in this group were markedly elevated relative to the WT+CA group (Figure S4), accompanied by synchronized upregulation of endothelial cell markers (CD31, CD34, CD105, and CD146) at the mRNA expression, whereas vWF mRNA expression showed significant downregulation. Protein-level validation confirmed these findings: Immunofluorescence staining indicated significantly higher CD31 protein content in the Bach1^-/-^+CA group compared to WT+CA controls (Fig. 3G, H). Western blot analysis further demonstrated substantial upregulation of VEGFA, VEGFR2, and endothelial markers (CD31, CD34, CD105, CD146) at the protein content, while vWF protein content was markedly reduced (Fig. 3I).

Three BACH1 siRNAs (siRNA 001, 002, and 003) were designed and synthesized to elucidate the role of BACH1 in mediating TCA-stimulated HUVECs (Figure S5 A, B). Compared with the siNC+TCA group, the siBACH1+TCA group exhibited significantly elevated mRNA expression levels of VEGFA and VEGFR2, accompanied by synchronous upregulation of endothelial cell markers (CD31, CD34, CD105, and CD146). In contrast, the mRNA expression of vWF showed a significant downregulation (Figure S5 C). These results were further confirmed by WB (Figure S5D). Additionally, the mRNA expression (Figure S5E) and protein content (Figure S5 F) of the proliferation-related marker PCNA were significantly upregulated in the TCA-treated group following BACH1 silencing.

### Bach1 deficiency promotes NAD^+^ transport to mitochondria

**Fig. 4:**
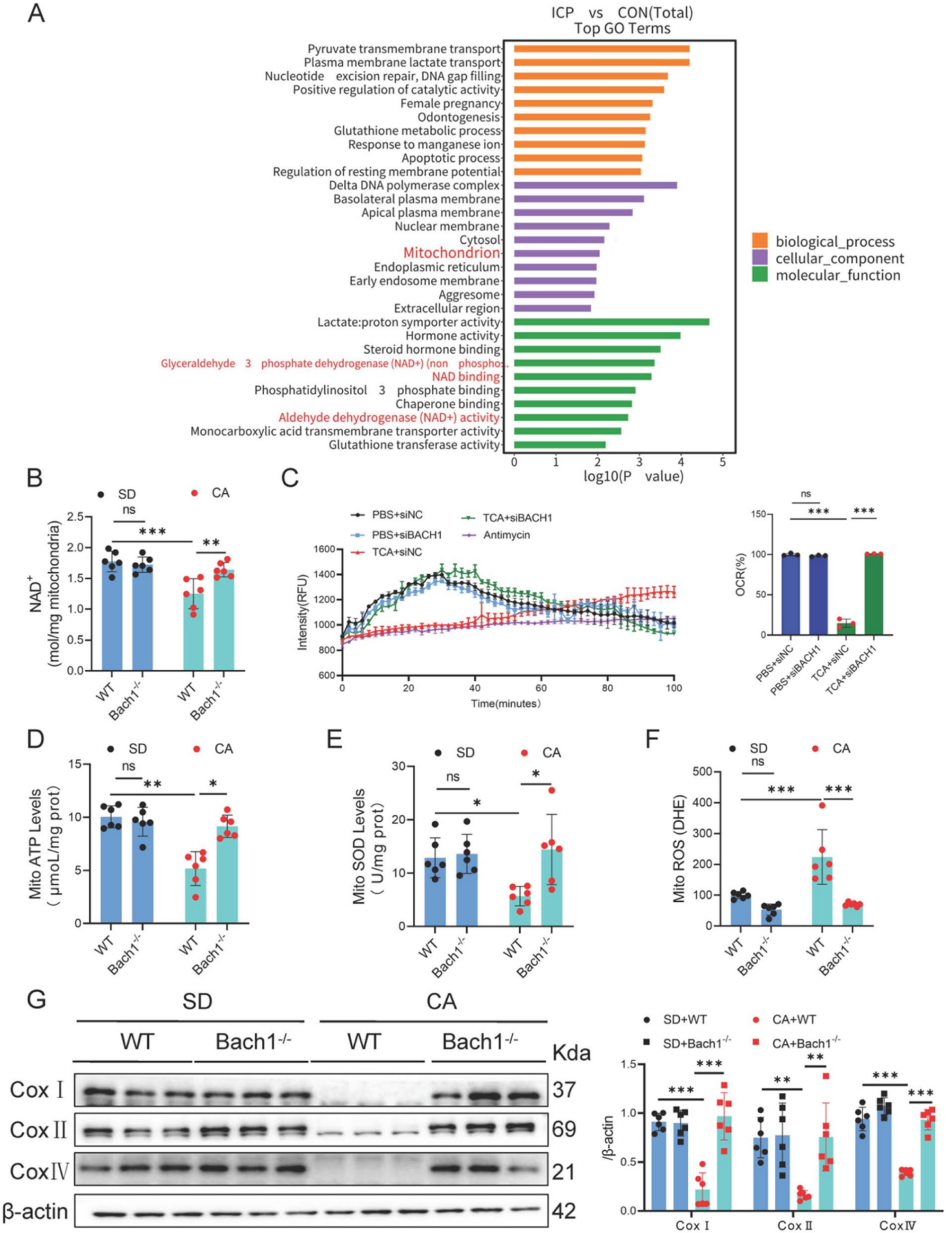
BACH1 knockout promotes NAD^+^ transport and activates mitochondrial electron transport chain in placenta of CA-induced mice. **A** Enrichment of DEGs between placentas of five normal pregnant women and five patients with ICP. **B** NAD^+^ concentration in mitochondria of placenta of WT or Bach1^-/-^ C57BL/6 mice treated with CA (*n* = 6). **C** OCR in HUVECs transfected with siBACH1 and stimulated with TCA. **D**–**F** ATP, SOD, and ROS levels in mitochondria of placenta of WT or Bach1^-/-^ C57BL/6 mice treated with CA (*n* = 6). **G** Western blotting for COXI, COXII, and COXIV in placenta of WT or Bach1^-/-^ C57BL/6 mice treated with CA (*n* = 6). One-way analysis of variance (ANOVA) followed by Tukey’s post-hoc analysis; ns: not significant, *p < 0.05, **p < 0.01, ***p < 0.001

Proteomic analysis identified 45 proteins that were upregulated and 45 proteins that were downregulated in the placentas of patients with ICP compared to normal individuals (Log2 fold change (log2 FC) > 0.263; Figure S6 A and B). Proteins associated with mitochondria and NAD^+^ were significantly enriched (Fig. 4A).

Comparative analysis of mitochondrial parameters in mouse placental tissue revealed that, relative to the WT+CA group, the Bach1^-/-^+CA group exhibited significantly elevated mitochondrial NAD^+^ content (Fig. 4B), ATP/SOD levels (Fig. 4D, E), and protein content of complexes I/II/IV (Fig. 4G), accompanied by reduced ROS levels (Fig. 4F). Similarly, in the HUVECs model, the siBACH1+TCA group demonstrated synergistic effects compared to the siNC+TCA group, including enhanced NAD^+^ content (Figure S6 C), increased OCR (Fig. 4C), elevated ATP/SOD levels (Figure S6D, E), upregulated content of complexes I/II/IV proteins (Figure S6G), and diminished ROS production (Figure S6 F).

### BACH1 deficiency promotes the transcriptional expression of SLC25 A51 in mice placentas

**Fig. 5:**
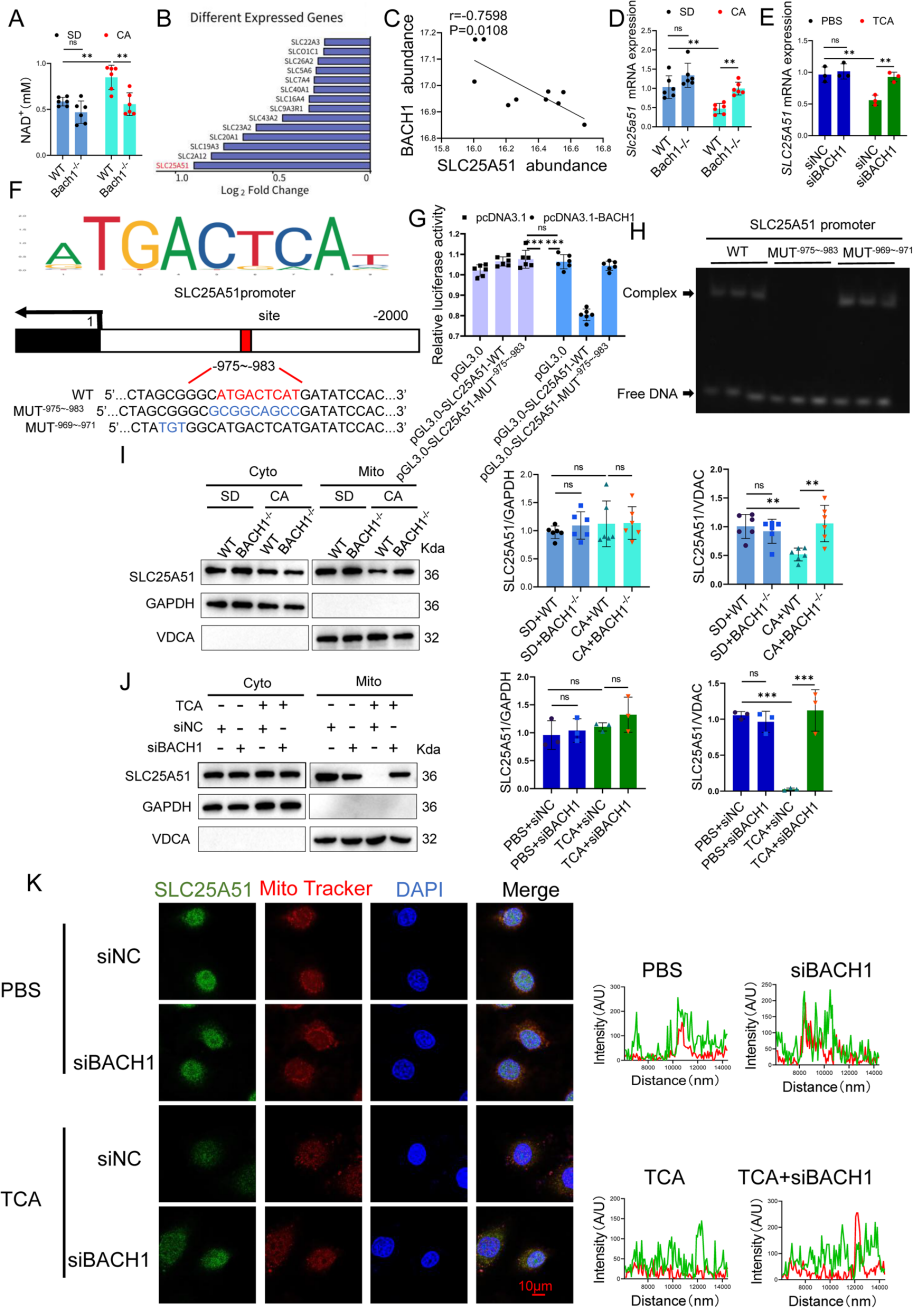
BACH1 deficiency promotes SLC25 A51 expression in TCA-induced HUVECs. **A** NAD^+^ content in mitochondria from placenta of WT or Bach1^-/-^ C57BL/6 mice treated with CA in vitro (*n* = 6). **B** *Differential SLC* expression in placental tissue from normal pregnant women and ICP patients. **C** Positive correlation between BACH1 and SLC25 A51 abundance in placenta of five normal pregnant women and five patients with ICP; Spearman correlation analysis. **D** *SLC25 A51* mRNA expression in siNC and siBACH1 HUVECs treated with TCA for 24 h (*n* = 3). **E** *SLC25 A51* mRNA expression in placenta of WT or Bach1^-/-^ C57BL/6 mice treated with CA for 7 days (*n* = 6). **F** BACH1 motif, BACH1-binding site, and mutation site on *SLC25 A51* promoter. **G** BACH1 localization on *SLC25 A51* promoter in HEK-293 T cells transfected with BACH1 overexpression or WT (SLC25 A51 WT) or mutant (SLC25 A51 MUT^−975~−983^) SLC25 A51 plasmids for 24 h. **H** Binding of BACH1 to *SLC25 A51* promoter in HUVECs. **I** Effects of *BACH1* gene knockout on SLC25 A51 protein abundance in mitochondria (Mito) and cytoplasm (Cyto) of placenta of WT and Bach1^-/-^ C57BL/6 mice treated with CA (*n* = 6). **J** Effect of *BACH1* silencing on SLC25 A51 protein abundance in Mito and Cyto in HUVECs with or without TCA treatment (*n* = 3). **K** Representative immunofluorescence images of HUVECs stained with anti-SLC25 A51 antibody (green), MitoTracker (red), and 4’,6-diamidino-2-phenylindole (DAPI; blue). Bars = 10 μm (*n* = 3); one-way analysis of variance (ANOVA) followed by Tukey’s post-hoc analysis; ns: not significant, **p < 0.01, ***p < 0.001

Mitochondria isolated from mouse placenta were co-incubated with NAD^+^ in vitro, and the NAD^+^ content in MiR05 medium was quantified. Compared to the WT+CA group, the Bach1^-/-^+CA group showed significantly reduced NAD^+^ levels in MiR05 (Fig. 5A). Proteomic analysis of the SLC family revealed SLC25 A51 as the most significantly downregulated SLC protein (Fig. 5B). BACH1 abundance exhibited a negative correlation with SLC25 A51 expression (Fig. 5C). Correspondingly, SLC25 A51 mRNA expression was significantly increased after BACH1 knockdown (Fig. 5D, E). Therefore, we hypothesize that BACH1 represses SLC25 A51 transcription, thereby affecting mitochondrial NAD^+^ transport.

To test this hypothesis, BACH1’s DNA motif and SLC25 A51 promoter binding site were obtained from the JASPAR database (http://jaspar.genereg.net/) (Fig. 5F), and mutations were introduced. Subsequent luciferase reporter gene assays revealed that BACH1 overexpression suppressed the activity of the WT-type *SLC25 A51* promoter (SLC25 A51-WT). Conversely, the activity of the *SLC25 A51* promoter in cells harboring mutations in the BACH1 binding site (SLC25 A51-MUT^−975~−983^) remained largely unchanged (Fig. 5G). These findings were corroborated by electrophoretic mobility shift assays (EMSA; Fig. 5H).

Herein, we isolated mitochondrial and cytoplasmic fractions from placental tissue. Compared with the WT+CA group, the Bach1^-/-^+CA group showed no changes in cytoplasmic SLC25 A51 protein content but exhibited a significant increase in mitochondrial SLC25 A51 protein content (Fig. 5I). Similarly, in HUVECs, the siBACH1+TCA group demonstrated unchanged cytoplasmic SLC25 A51 protein content but notably elevated mitochondrial SLC25 A51 protein content compared to the siNC+TCA group (Fig. 5J). These observations were further corroborated by cellular immunofluorescence analysis (Fig. 5K).

### SLC25A51 promotes the transport of NAD^+^ to mitochondria in TCA-induced HUVECs

**Fig. 6:**
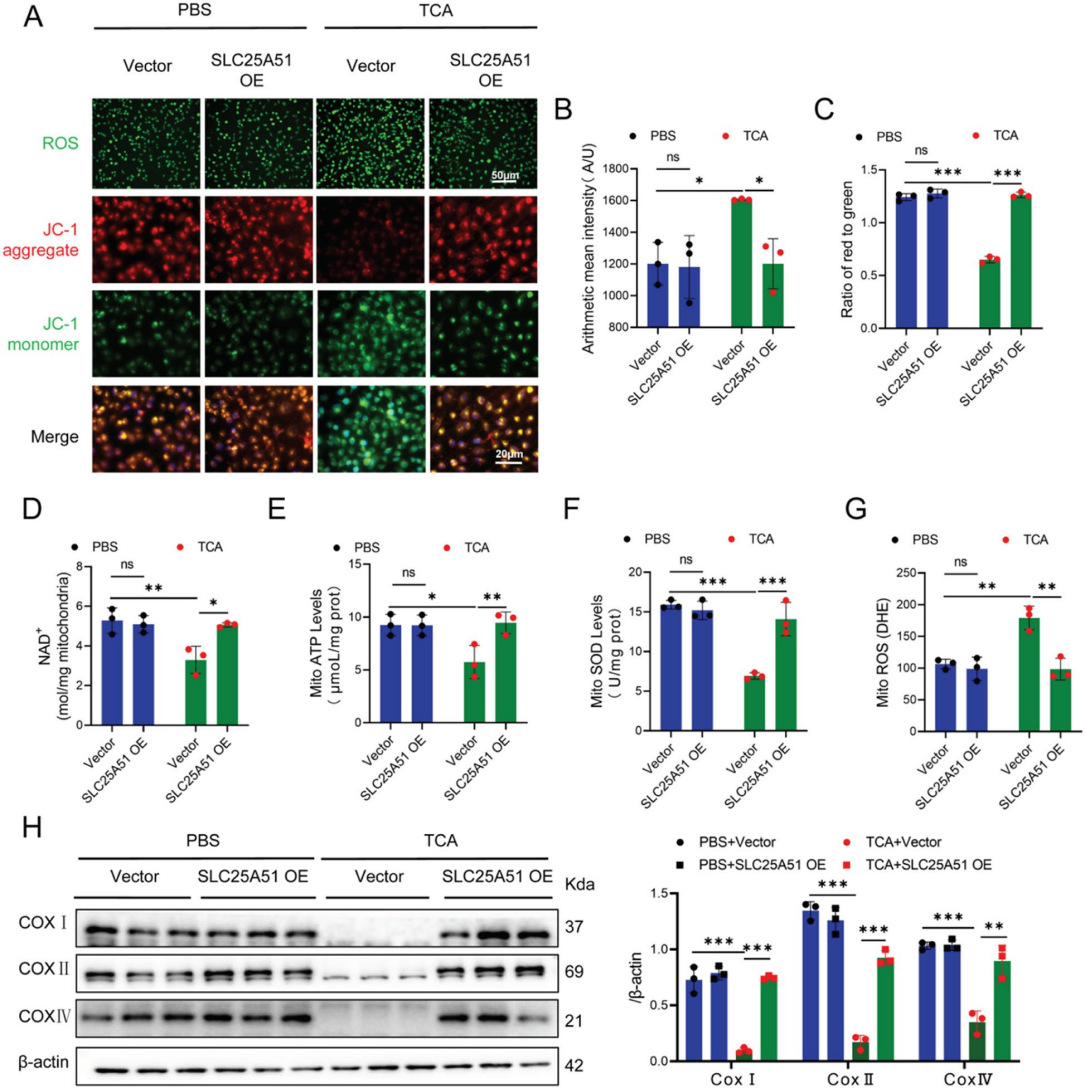
SLC25 A51 Overexpression promotes NAD^+^ transport to improve mitochondrial respiratory function in HUVECs treated with TCA. **A**–**C** Immunostaining for ROS (green), JC-1 aggregate (red), and JC-1 monomer (green) in Vector or SLC25 A51-overexpressing (OE) HUVECs treated with TCA for 24 h (scale bar: 50 μM; *n* = 3). **D**–**G** NAD^+^, ATP, SOD, and ROS concentrations in mitochondria of Vector or SLC25 A51 OE HUVECs treated with TCA for 24 h (*n* = 3). **H** Western blotting of COXI, COXII, and COXIV in Vector or SLC25 A51 OE HUVECs treated with TCA for 24 h (*n* = 3). One-way analysis of variance (ANOVA) followed by Tukey’s post-hoc analysis; ns: not significant, *p < 0.05, **p < 0.01, ***p < 0.001

HUVECs were transfected with SLC25 A51 OE plasmids (Figure S7 A–B). The immunofluorescence results demonstrated that compared to the Vector+TCA group, the SLC25 A51 OE+TCA group exhibited significantly reduced ROS levels (Fig. 6A, B) and markedly elevated JC-1 fluorescence intensity (Fig. 6A, C). Furthermore, in comparison with the Vector+TCA group, the SLC25 A51 OE+TCA group showed significantly increased mitochondrial NAD^+^ content (Fig. 6D), elevated ATP/SOD levels (Fig. 6E, F), enhanced protein content of complexes I/II/IV (Fig. 6H), along with decreased ROS production (Fig. 6G).

The HUVECs stimulated with TCA were transfected with shSLC25 A51 lentivirus (Figure S8 A). Compared to the shNC+TCA group, the shSLC25 A51+TCA group exhibited significantly increased mitochondrial NAD^+^ content (Figure S8B), elevated ATP/SOD levels (Figure S8 C, D), and enhanced complex I/II/IV protein content (Figure S8 F), while showing decreased ROS levels (Figure S8E).

### SLC25A51 downregulation reverses the effect of BACH1 deficiency on TCA-stimulated HUVEC proliferation

**Fig. 7:**
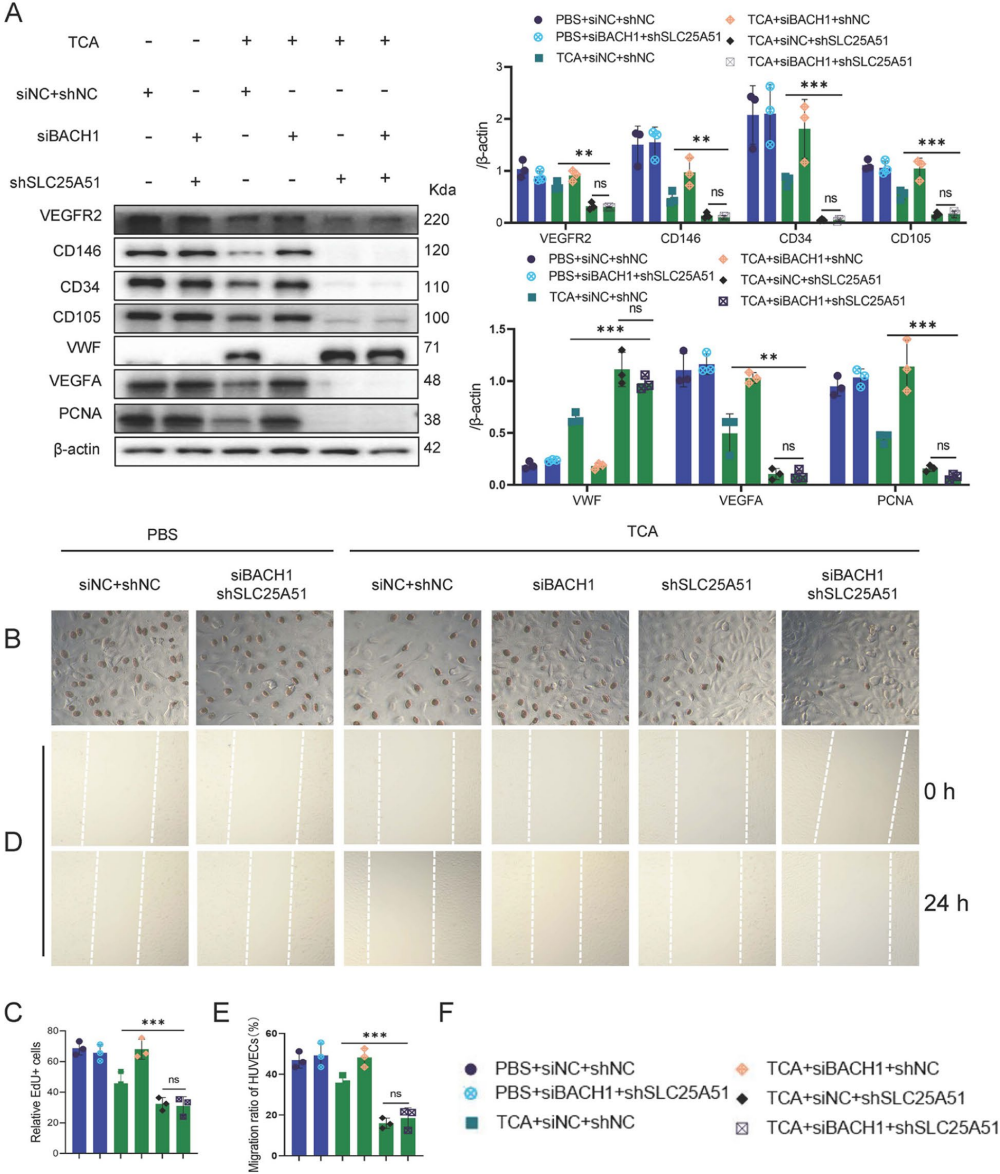
Suppression of SLC25 A51 expression reversed effect of silencing BACH1 on proliferation of HUVECs stimulated by TCA. **A** Western blotting of VEGFR2, CD146, CD34, CD35, vWF, VEGFA, and PCNA in siNC, shNC, siBACH1, and shSLC25 A51 HUVECs treated with TCA for 24 h (*n* = 3). **B**, **C** Relative EdU+ cells in siNC, shNC, siBACH1, and shSLC25 A51 HUVECs treated with TCA for 24 h (*n* = 3). **D**, **E** Wound healing assay of siNC, shNC, siBACH1, and shSLC25 A51 HUVECs treated with TCA for 24 h (*n* = 3). **F** Different colors represent corresponding groups. One-way analysis of variance (ANOVA) followed by Tukey’s post-hoc analysis; ns: not significant, *p < 0.05, **p < 0.01, ***p < 0.001

To determine whether SLC25 A51 is the essential mitochondrial membrane protein mediating BACH1-regulated NAD^+^ transport into mitochondria, we transfected siBACH1 into HUVECs stably expressing shSLC25 A51. Compared with the siBACH1+TCA group, the siBACH1+shSLC25 A51+TCA group exhibited significantly downregulated protein content of VEGFA, VEGFR2, and endothelial markers (CD31, CD34, CD105, CD146), while vWF protein content were markedly upregulated (Fig. 7A). Similarly, EdU staining revealed a significant reduction in the proportion of EdU+ proliferating cells (Fig. 7B, C). Compared to the siBACH1+TCA group, the siBACH1+shSLC25 A51+TCA group demonstrated impaired HUVECs migration (Fig. 7D, E).

### BAHC1 inhibitor HPPE promotes TCA-induced HUVECs proliferation

**Fig. 8:**
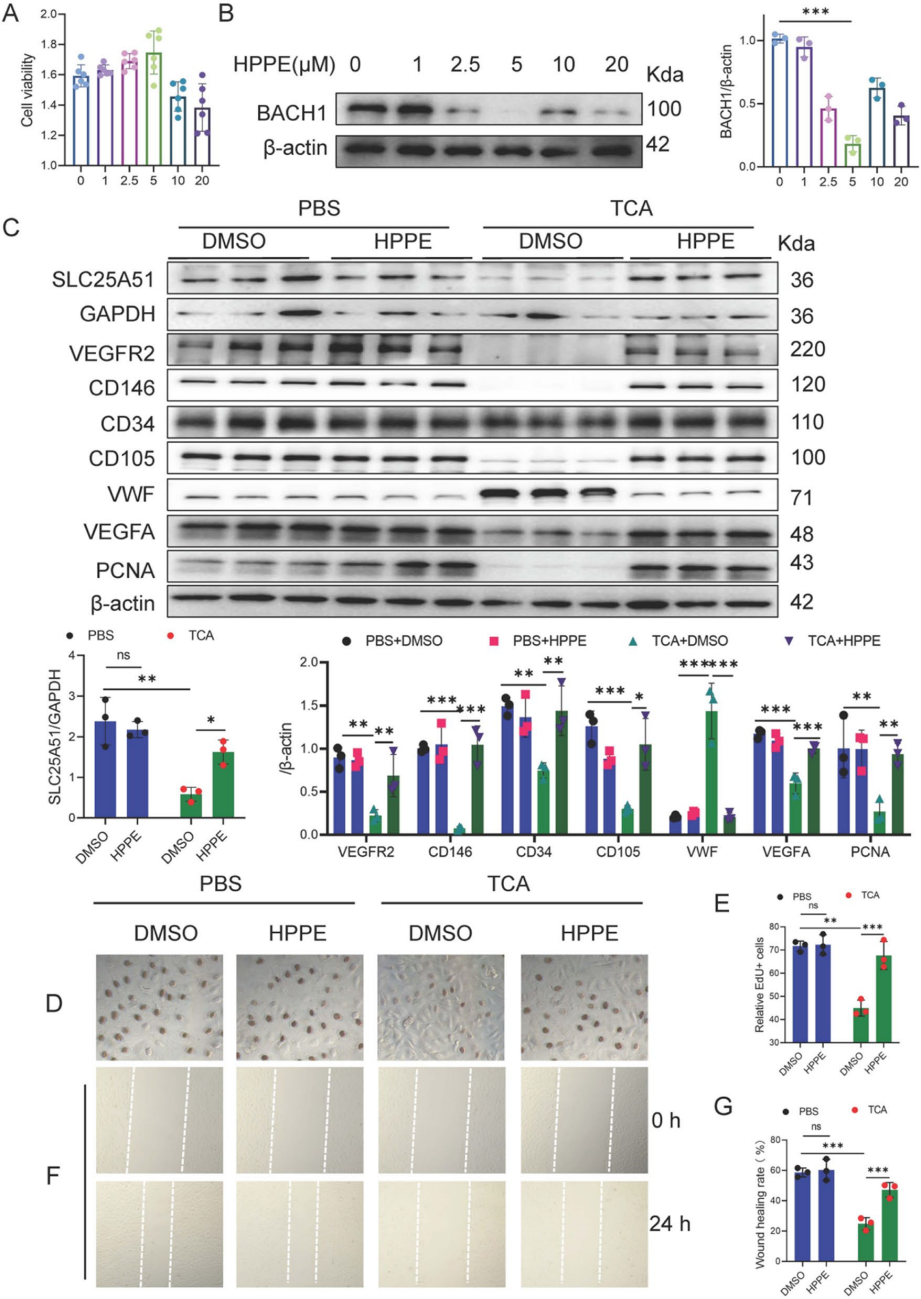
BAHC1 inhibitor HPPE promotes TCA-induced HUVECs proliferation. **A** Viability of HUVECs treated with different concentrations of HPPE (0, 1, 2.5, 5, 10, and 20 μM; *n* = 3). **B** Western blotting results for BACH1 in HUVECs treated with different concentrations of HPPE (0, 1, 2.5, 5, 10, and 20 μM); bar graph represents quantification of gray value (*n* = 3). **C** Western blotting results for SLC25 A51, VEGFR2, CD146, CD34, CD105, vWF, VEGFA, and PCNA in HUVECs treated with HPPE (5 μM; *n* = 3). **D**, **E** Relative number of EdU+ cells among HUVECs treated with HPPE (5 μM; *n* = 3). **F**, **G** Wound healing assay of HUVECs treated with HPPE (5 μM) 24 h after challenge (*n* = 3). One-way analysis of variance (ANOVA) followed by Tukey’s post-hoc analysis; ns: not significant, *p < 0.05, **p < 0.01, ***p < 0.001

Recent studies have identified HPPE as a promising inhibitor of BACH1 for treating Parkinson’s disease (Ahuja et al. 2021). To investigate its potential therapeutic effect in ICP, HUVECs were treated with various concentrations of HPPE. BACH1 expression was minimal at an HPPE concentration of 5 μM, with an inhibition efficiency of 82.22% (Fig. 8A, B). Compared to the DMSO+TCA group, the HPPE+TCA group showed significant upregulation in both SLC25 A51 mRNA expression (Figure S9 A) and protein content (Fig. 8C). Additionally, mRNA expression (Figure S9B) and protein content (Fig. 8C) levels of VEGFA, VEGFR2, and endothelial markers (CD31, CD34, CD105, CD146) were markedly increased, whereas vWF mRNA expression (Figure S9B) and protein content (Fig. 8C) were significantly downregulated. Similarly, PCNA mRNA expression (Figure S9 C) and protein content (Fig. 8C) were notably elevated. In comparison to the DMSO+TCA group, the HPPE+TCA group enhanced the migration of HUVECs (Human Umbilical Vein Endothelial Cells) (Fig. 8F, G).

## Discussion

The results of this study highlight the role of BACH1 in regulating placental angiogenesis in ICP. Specifically, BACH1 inhibits the transcription of SLC25 A51, a mitochondrial transporter of NAD^+^ in endothelial cells. This suppression impedes NAD^+^ translocation into the mitochondria. Therefore, mitochondrial respiratory function is impaired and the number of placental vessels is reduced. Therefore, BACH1 may act as a regulator of mitochondrial respiratory function, suggesting that it can provide a theoretical basis for the treatment of ICP placental damage.

Our study revealed that BACH1 knockout alleviates placental damage in ICP. An increasing body of research has elucidated the broad expression profile and multifaceted regulatory roles of BACH1, a transcriptional repressor, across human tissues (Wei et al. 2025). Specifically, BACH1 modulates the development and functionality of innate and adaptive immune systems in the context of non-gestational diseases (So et al. 2012). Additionally, it influences diverse biological processes, including apoptosis, angiogenesis, lymphangiogenesis, oxidative stress injury, embryonic stem cell senescence and pluripotency in non-gestational diseases (Lv et al. 2025; Folkert et al. 2024; Cohen et al. 2020; Padilla et al. 2021; Niu et al. 2021; Chu et al. 2022; Huang et al. 2025). The placenta serves as a critical interface for maternal-fetal substance exchange (He et al. 2024a, b). Recent studies have demonstrated that placental damage primarily manifests as reductions in fetal weight, placental weight, placental diameter, fetal-to-placental weight ratio, and capillary density, collectively contributing to placental dysfunction (Du et al. 2025; Xie et al. 2025; Wang et al. 2025a, b). Our findings indicate that BACH1 knockout significantly ameliorates ICP-induced placental damage.

Disruptions in placental angiogenesis result in decreased vascularity, vasoconstriction, and elevated vascular resistance, collectively impairing blood and oxygen supply to the placenta and ultimately leading to placental dysfunction (Yang et al. 2024). Recent investigations have underscored the importance of placental vasculature in ICP, with associated biomarkers explored for ICP diagnosis (Du et al. 2014; Wang et al. 2021a, b). Recent studies have revealed that angiogenesis is primarily driven by vascular endothelial growth factor (VEGF) and its receptor VEGFR (Choi et al. 2025). Activation of the VEGFA/VEGFR2 signaling pathway has been shown to enhance the migration and proliferation of HUVECs (Chen et al. 2025a, b). Research indicates a significant increase in endothelial cell markers within primary intraosseous vascular endothelial cells of Rarres2^-/-^ mice, further suggesting enhanced neovascularization (Wang et al. 2025a, b). Reduced levels of vWF can alleviate endothelial cell dysfunction (Zheng et al. 2024). Our study demonstrates that BACH1 knockout activates the VEGFA/VEGFR2 signaling pathway, upregulates endothelial cell marker expression, and decreases vWF levels. These findings suggest that BACH1 may indirectly regulate VEGFR2 stability through its interacting partners (e.g., VEGFC) (Cohen et al. 2020). Previous studies have confirmed that reduced VEGFR2 protein levels correlate with diminished p-VEGFR2 (phosphorylated VEGFR2) (Chen et al. 2025a, b). Notably, Yuan Gu et al. (2025) demonstrated that the anti-angiogenic effect of chloroquine is mediated through downregulation of ERK phosphorylation, which is at least partially attributed to VEGFR2 degradation. Collectively, these evidences indicate that BACH1 inhibition may exert significant pro-angiogenic effects.

Mitochondrial oxidative stress and dysfunction have emerged as pivotal factors in angiogenesis regulation (Liu et al. 2024). The bioinformatics analysis of the current study confirms the significant role of mitochondria and NAD^+^-associated processes in ICP. BACH1 has been extensively studied in the context of antioxidative responses (Liu et al. 2024). A subset of BACH1-target genes is related to mitochondrial function, encompassing a wide range of processes from mitochondrial transcription and biogenesis to bioenergetics (Ahuja et al. 2022). Furthermore, BACH1 negatively modulates the expression of several genes integral to the mitochondrial ETC, leading to increased mitochondrial acidification and decreased respiratory function (Lee et al. 2019). Therefore, our data indicate that BACH1 knockout can enhance the mitochondrial transport of NAD^+^, subsequently mitigating mitochondrial oxidative stress in the ICP placenta.

The SLC25 subfamily, which belongs to the extensive solute carrier (SLC) family, comprises a diverse array of mitochondrial inner membrane transporters that are indispensable for various metabolic pathways and cellular functions. While most SLC family members act as strict anti-exchangers of chemically related substrates, the precise roles of others remain elusive (Luongo et al. 2020; Ruprecht et al. 2020; Fiermonte et al. 2004; Wang et al. 2021a, b). The current bioinformatics analysis revealed significant SLC25 A51 downregulation, with a notable absence of SLC25 A51-mediated repression of NAD^+^ transport into mitochondria. In fact, it has been reported that SLC25 A51 overexpression enhances NAD^+^ transport into mitochondria, increases OCR, mitochondrial respiratory chain complex activities (I-V), SOD levels, and ATP production, while simultaneously suppressing ROS generation (Bai et al. 2022). Therefore, promoting the expression of SLC25 A51 may enhance mitochondrial respiratory function.

BACH1 has been characterized as an oxygen- and redox-sensitive transcription factor that governs tumor angiogenesis and vascular patterning, rendering tumors susceptible to anti-angiogenic therapies (Wang et al. 2023). Additionally, BACH1 modulates the expression of genes encoding components of the mitochondrial electron transport chain (Yang et al. 2022a, b). The precise mechanisms by which BACH1 regulates mitochondrial function and placental angiogenesis in the context of ICP remain elusive. However, our findings reveal that BACH1 knockout promotes the transcription of SLC25 A51, thereby improving mitochondrial respiratory function.

Endothelial cell proliferation is influenced by signaling cascades and intricate intracellular metabolic pathways (Qian et al. 2023). Herein, SLC25 A51 expression was upregulated in the placenta of BACH1^-/-^ mice, and its consequences on mitochondrial respiratory function were elucidated. In ICP, we observed that reduced expression of SLC25 A51 impairs mitochondrial NAD^+^ transport, subsequently leading to decreased activity of respiratory chain complexes I/II/IV.

Certainly, there are certain limitations in our research. For the fetus, ICP can lead to more adverse outcomes than the mother, including preterm birth, amniotic fluid contamination, neonatal depression, respiratory distress syndrome, and increased risk of stillbirth (Li et al. 2025). However, this study has certain limitations in investigating fetal growth and development. In future studies, we will focus specifically on fetal growth and development. Secondly, we acknowledge that we have not utilized endothelial cell-specific Bach1 knockout mice. Finally, this study did not use single-cell sequencing to screen for PVECs, nor did it isolate PVECs.

This study delineated a novel mechanism underlying placental angiogenesis in ICP. Specifically, the upregulation of BACH1 is implicated in the pathogenesis of placental angiogenesis in ICP. BACH1 binds to the *SLC25 A51* promoter as a transcriptional repressor. This disrupts the transport of NAD^+^ into mitochondria and exacerbates mitochondrial oxidative stress. Furthermore, regulating the activity of BACH1 may potentially provide therapeutic benefits by modulating placental angiogenesis that is associated with ICP.

## Supplementary Information

Supplementary Material 1

## Data Availability

No datasets were generated or analysed during the current study.

## Abbreviations

SD: Standard diet
E2: 17α-ethinyl estradiol
CA: Cholic acid
BACH1: BTB and CNC homology 1
VEGFR2: Kinase Insert Domain Receptor;
CD31: Platelet endothelial cell adhesion molecule-1, PECAM-1
CD164: Cluster of Differentiation 164
CD34: Highly glycosylated type I transmembrane glycoprotein
CD105: Endoglin
vWF: von Willebrand Factor
VEGFA: Vascular Endothelial Growth Factor A
TBA: Total bile acid
AST: Aspartate Aminotransferase
ALT: Alanine Aminotransferase
COX I: Cytochrome c Oxidase Subunit I
COX II: Cyclooxygenase-2
COX IV: Cytochrome c oxidase subunit IV
NAD^+^: Nicotinamide Adenine Dinucleotide
ROS: Reactive Oxygen Species
ATP: Adenosine Triphosphate
SOD: Superoxide Dismutase
JC-1: J-aggregate forming fluorescent dye
PCNA: Proliferating Cell Nuclear Antigen
SLC25 A51: Solute carrier family 25 member 51
SLC25 A53: Solute carrier family 25 member 53
PGF: Placental Growth Factor
IGF1: Insulin-like Growth Factor 1
GLUT1: Glucose Transporter Type 1
PBS: Phosphate-buffered saline
RT-qPCR: real-time quantitative PCR
