# Supplementary material for: BACH1 deficiency improves placental angiogenesis via SLC25A51-mediated mitochondrial NAD+ transport in intrahepatic cholestasis of pregnancy
Source: Mol Med. 2025 May 1;31:162. doi: 10.1186/s10020-025-01215-4 (PMC12044804; doi:10.1186/s10020-025-01215-4)
Supplement: Supplementary file 1 — Supplementary Material 1 [file 10020_2025_1215_MOESM1_ESM.docx]

**BACH1 deficiency improves placental angiogenesis via SLC25A51-mediated mitochondrial NAD^+^ transport in intrahepatic cholestasis of pregnancy**

Shengpeng Li^1, #^, Weiying Zhu^2, #^, Zhixuan Xing^1^, Dan Chen^1^, Huimin Zhao^1^, Yanli Zhang^1^, Wenlong Zhang^1^, Jiaojiao Sun^1^, Yaxian Wu^1^, Ling Ai^2, *^, Qingfeng Pang^1, *^

1 Wuxi School of Medicine, Jiangnan University, 1800 Lihu Avenue, Wuxi, 214122, Jiangsu province, PR China

^2^ Department of Obstetrics, Maternity and Child Health Care Affiliated Hospital, Jiaxing University, NO.2468 East Central Road, South Lake District, Jiaxing, 314000, China

**Lead contact:**

*Corresponding authors: E-mail address: qfpang@jiangnan.edu.cn (Qingfeng Pang); 13736836830@163.com (Ling Ai)

**Table S1.** Basic medical characteristics of subjects recruited in this study

| **Characteristic** | **Normal**  **(*n* = 36)** | **ICP**  **(*n* = 33)** | ***p*-value** |
| --- | --- | --- | --- |
| Age (years) | 28.81 ± 3.92 | 29.03 ± 4.10 | 0.718 |
| TBA (μmol/L) | 4.99 ± 3.1 | 25.58 ± 20.56 | 0.000 |
| AST (U/L) | 18.14 ± 10.81 | 70.01 ± 156.74 | 0.000 |
| ALT (U/L) | 11.47 ± 15.55 | 65.64 ± 122.62 | 0.000 |
| CHO (mmol/L) | 6.24 ± 1.97 | 6.00 ± 1.29 | 0.000 |
| Placental volume (cm^3^) | 1,008.76 ± 331.02 | 976.28 ± 314.79 | 0.683 |
| Gestational weight (kg) | 67.95 ± 8.09 | 66.1 ± 9.13 | 0.778 |
| Gestational age at delivery (weeks) | 39.02 ± 1.49 | 37.12 ± 3.52 | 0.005 |
| Gestational height (cm) | 159.75 ± 4.42 | 157.55 ± 5.27 | 0.067 |
| Birth weight (kg) | 3.32 ± 0.46 | 3.05 ± 0.82 | 0.001 |

^a^ TBA, total bile acids;^b^ AST, aspartate aminotransferase;^c^ ALT, alanine aminotransferase;^d^ CHO, total cholesterol

**Table S2.** Primer sequences for BACH1 and SLC25A51 siRNA

| **Target gene** | **Primer** | **Primer sequences (5′–3′)** |
| --- | --- | --- |
| *BACH1-siRNA 001* | Forward | GCAAUGGAACCUGAAGAAATT |
|  | Reverse | UUUCUUCAGGUUCCAUUGCTA |
| *BACH1-siRNA 002* | Forward | GGAGACAGUGAAUCCUGUUTT |
|  | Reverse | AACAGGAUUCACUGUCUCCTT |
| *BACH1-siRNA 003* | Forward | GUAGAGCUGCAUUGUGAAATT |
|  | Reverse | UUUCACAAUGCAGCUCUACAA |
| *SLC25A51-siRNA 001* | Forward | CCAUUGAUGCAGAAGACAATT |
|  | Reverse | UUGUCUUCUGCAUCAAUGGTT |
| *SLC25A51-siRNA 002* | Forward | GCAAUACUUCAGUUGAGAATT |
|  | Reverse | UUCUCAACUGAAGUAUUGCTT |
| *SLC25A51-siRNA 003* | Forward | GAGUUCUUGUUAAAGGUUATT |
|  | Reverse | UAACCUUUAACAAGAACUCTT |

**Table S3.** SLC25A51 shRNA sequences

| **Target gene** | **Sequence (5′–3′)** |
| --- | --- |
| *siSLC25A51 001* | CCGGGCACTTATGTTTGGTCTGTATCTCGAGATACAGACCAAACATAAGTGCTTTTTG |
| *siSLC25A51 002* | CCGGGCAACTTATGAGTTCTTGTTACTCGAGTAACAAGAACTCATAAGTTGCTTTTTGTGT |
| *siSLC25A51 003* | CCGGGCACTGAAATGTCATGGAATTCTCGAGAATTCCATGACATTTCAGTGCTTTTTG |
| *shGFP* | GCAAGCTGACCCTGAAGTTCAT |

**Table S4.** Primary antibodies used in this study

| **Antibodies** | **Source** | **Cat No.** | **Location** | **Dilution** |
| --- | --- | --- | --- | --- |
| Anti-CD31 | Cell Signaling Technologies | 77699s | Massachusetts, USA | 1:1,000 |
| Anti-KDR | Proteintech | 26415-1-AP | Wuhan, China | 1:1,000 |
| Anti-VEGFA | Proteintech | 66828-1-Ig | Wuhan, China | 1:1,000 |
| Anti-BACH1 | Proteintech | 80593-1-RR | Wuhan, China | 1:1,000 |
| Anti-PCNA | Proteintech | 10205-2-AP | Wuhan, China | 1:1,000 |
| Anti-CD146 | Proteintech | 17564-1-AP | Wuhan, China | 1:1,000 |
| Anti-CD34 | Proteintech | 14486-1-AP | Wuhan, China | 1:1,000 |
| Anti-CD105 | Proteintech | 10862-1-AP | Wuhan, China | 1:1,000 |
| Anti-VWF | Proteintech | 11778-1-AP | Wuhan, China | 1:1,000 |
| Anti-COXI | Abcam | 109025 | Massachusetts, USA | 1:1,000 |
| Anti-COXII | Abcam | 179800 | Massachusetts, USA | 1:1,000 |
| Anti-COXIV | Abcam | 202554 | Massachusetts, USA | 1:1,000 |
| Anti-SLC25A51 | Cusabio | CSB-PA875649LA01HU | Beijing, China | 1:1,000 |
| Anti-β-actin | Abcam | 263962 | Massachusetts, USA | 1:5,000 |
| Anti-GAPDH | Proteintech | 10494-1-AP | Wuhan, China | 1:5,000 |

**Table S5.** Primers for Real-Time Quantitative PCR Analysis

| **Gene** | **Forward (3′–5′)** | **Reverse (5′–3′)** |
| --- | --- | --- |
| *Human-BACH1* | TCTGAGTGAGAACTCGGTTTTTG | CGCTGGTCATTAAGGCTGAGTAA |
| *Mouse-Bach1* | AGAGTGCGGTATTTGCCTACG | TCAGTCTGGCCTACGATTCTC |
| *Human-SLC255A1* | AGGTCCTCTTTCGACAACAGC | ACCAAACATAAGTGCAAGCGTA |
| *Mouse-Slc25a51* | ATGATGGACTCCGAAGCACAT | GGGTAAGTGATCGCCACGTT |
| *Human-KDR* | GGCCCAATAATCAGAGTGGCA | CCAGTGTCATTTCCGATCACTTT |
| *Mouse-Kdr* | TTTGGCAAATACAACCCTTCAGA | GCTCCAGTATCATTTCCAACCA |
| *Human-CD31* | AACAGTGTTGACATGAAGAGCC | TGTAAAACAGCACGTCATCCTT |
| *Mouse-Cd31* | ACGCTGGTGCTCTATGCAAG | TCAGTTGCTGCCCATTCATCA |
| *Human-CD34* | CTACAACACCTAGTACCCTTGGA | GGTGAACACTGTGCTGATTACA |
| *Mouse-Cd34* | CTGGGTAGCTCTCTGCCTGAT | TGGTAGGAACTGATGGGGATATT |
| *Human-CD164* | ACCCGAACGTGACGACTTTAG | CGTGTTCCCCACTTGACAATC |
| *Mouse-Cd164* | AGAAACCTGTGCGAGCTTCAA | CACAAGTCAGTGCGGTTCAC |
| *Human-CD105* | TGCACTTGGCCTACAATTCCA | AGCTGCCCACTCAAGGATCT |
| *Mouse-Cd105* | AGGGGTGAGGTGACGTTTAC | GTGCCATTTTGCTTGGATGC |
| *Human-VEGFA* | AGGGCAGAATCATCACGAAGT | AGGGTCTCGATTGGATGGCA |
| *Mouse-Vegfa* | CTGCCGTCCGATTGAGACC | CCCCTCCTTGTACCACTGTC |
| *Human-VWF* | CCGATGCAGCCTTTTCGGA | TCCCCAAGATACACGGAGAGG |
| *Mouse-Vwf* | CTCTTTGGGGACGACTTCATC | TCCCGAGAATGGAGAAGGAAC |
| *Human-PCNA* | CCTGCTGGGATATTAGCTCCA | CAGCGGTAGGTGTCGAAGC |
| *Mouse-Pcna* | TTGCACGTATATGCCGAGACC | GGTGAACAGGCTCATTCATCTCT |
| *Human-SLC25A53* | TTGGGGCCGTTTCCAACTTTA | CCCGGTAGAAGTATTGAGGACC |
| *Mouse-Slc25a53* | ACACCCTTGGGGCGATTTC | GCTGCCGGAACACAACCTTAT |
| *Mouse-Pgf* | AGTGGAAGTGGTGCCTTTCAA | GTGAGACACCTCATCAGGGTA |
| *Mouse-Igf1* | CACATCATGTCGTCTTCACACC | GGAAGCAACACTCATCCACAATG |
| *Mouse-Glut1* | TCAAACATGGAACCACCGCTA | AAGAGGCCGACAGAGAAGGAA |


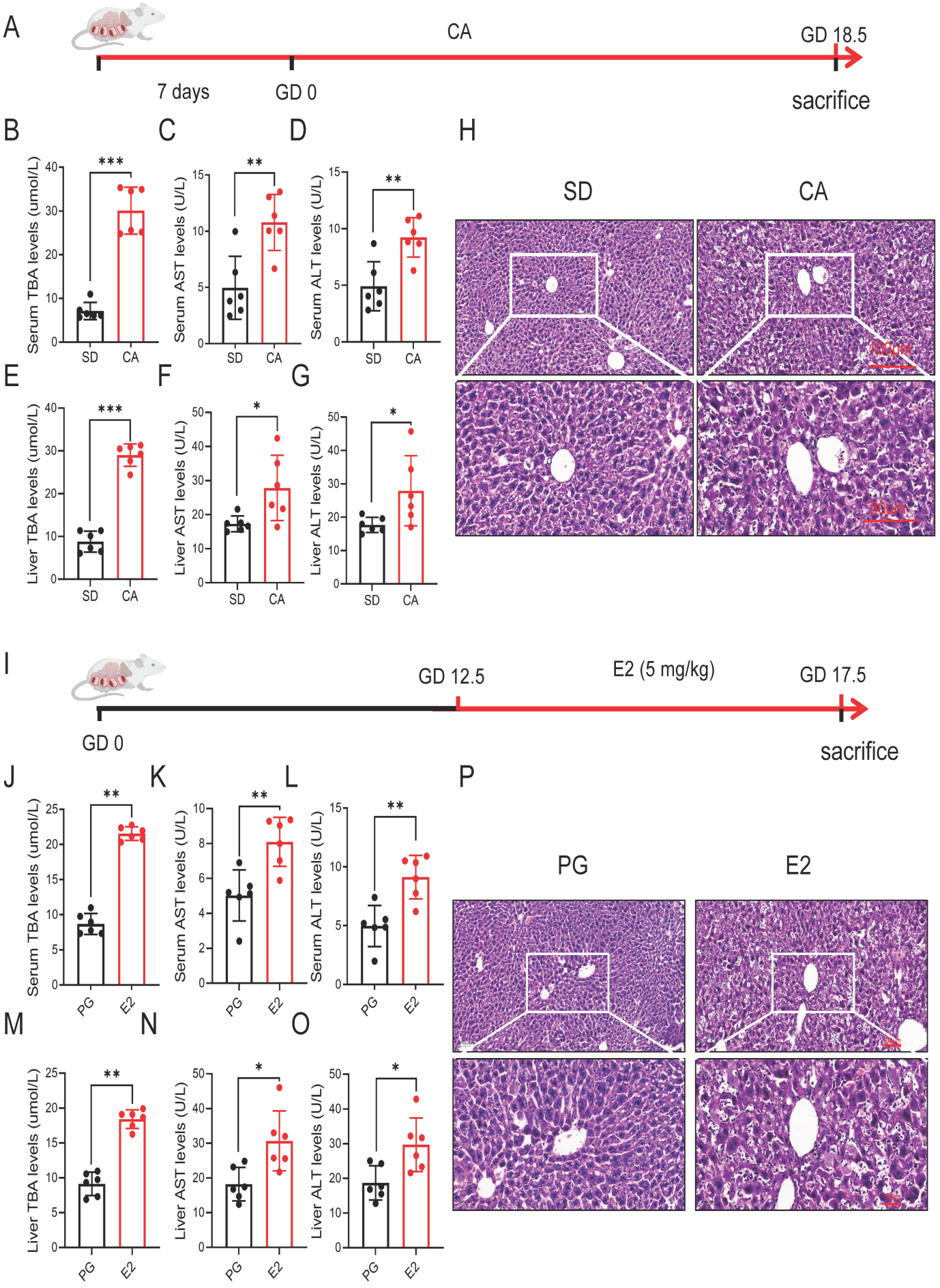


Fig. S1. Construction of intrahepatic cholestasis of pregnancy (ICP) mouse model.

(A) Mouse ICP model constructed using cholic acid (CA). (B–D) Serum total bile acid (TBA), aspartate aminotransferase (AST), and alanine aminotransferase (ALT) in standard diet (SD) and CA groups (*n* = 6). (E–G) TBA, AST, and ALT content in livers of SD and CA groups (*n* = 6). (H) Hematoxylin and eosin (H&E) staining of livers in SD and CA groups. (scale bar: 100 μM, 50 μM). (I) Mouse ICP model constructed by 17α-ethinylestradiol (E2) intraperitoneal injection. (J–L) Serum TBA, AST, and ALT content in propylene glycol (PG) and E2 groups (*n* = 6). (M–O) TBA, AST, and ALT content in livers of PG and E2 groups (*n* = 6). (P) H&E staining of livers in PG and E2 groups (scale bar: 100 μM, 50 μM). Unpaired, two-tailed Student’s *t*-test; **p* < 0.05, ***p* < 0.01, and ****p* < 0.001.


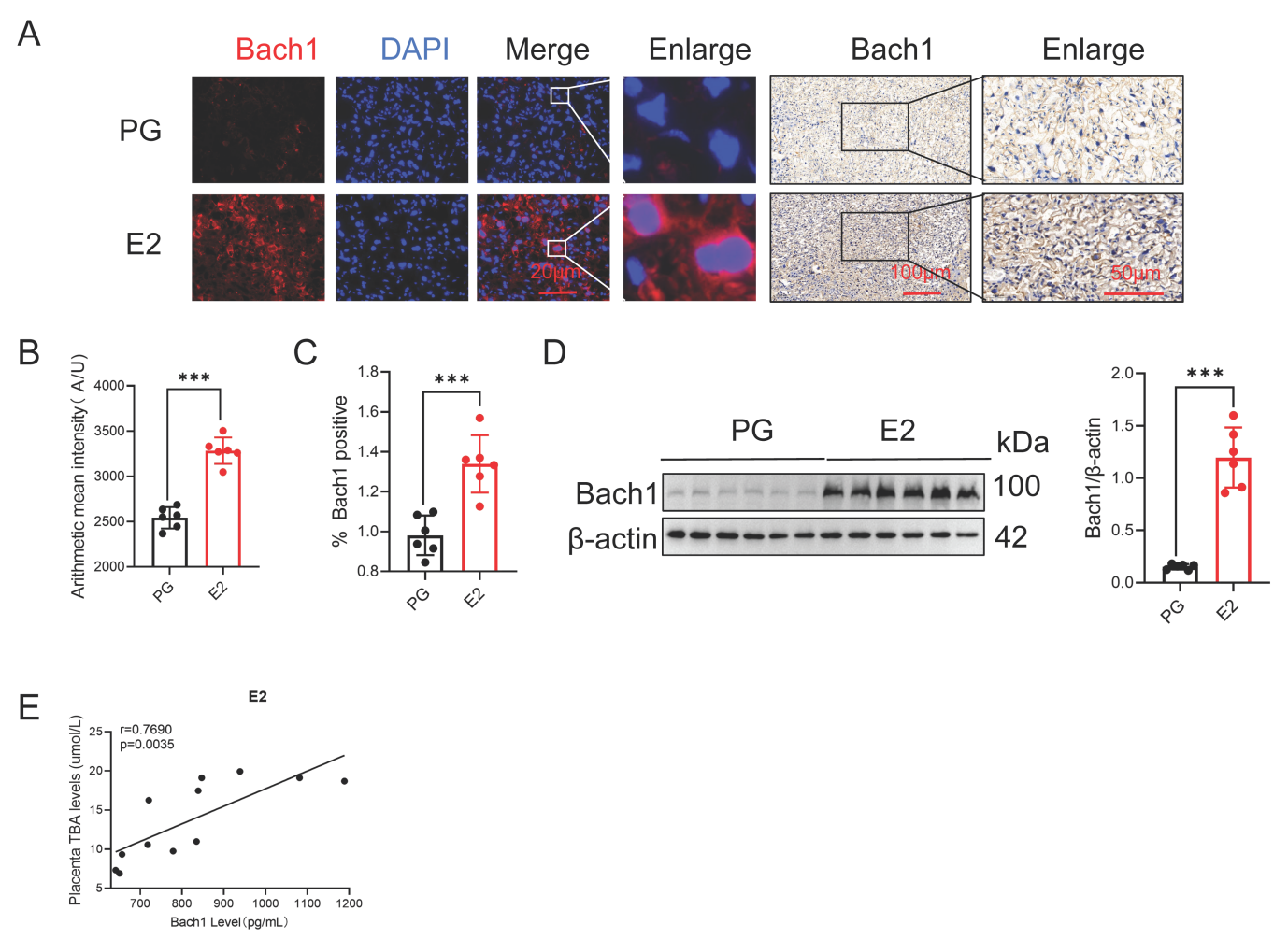


Fig. S2. BACH1 is highly expressed in 17α-ethinylestradiol (E2)-induced mouse placenta.

**(**A–C) ICP mouse model immunostained for BACH1 protein (red) and DAPI (blue); scale bar: 20 μM, 100 μM, 50 μM; *n* = 6). (D) Western blotting results for BACH1 in ICP mouse model (*n* = 6); unpaired, two-tailed Student’s *t*-test. (E) Strong positive correlation between BACH1 and TBA content in placentas of wild-type (WT) mice treated with E2; Spearman correlation; ****p* < 0.001.


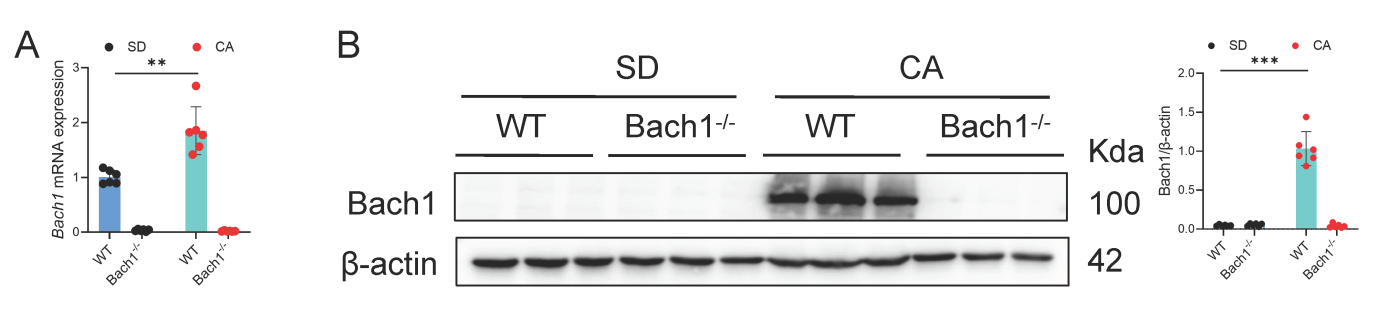


Fig. S3. Establishment of Bach1-knockout mice.

(A) *Bach1* mRNA in placentas of wild-type (WT) or Bach1^-/-^ C57BL/6 mice treated with CA (*n* = 6). (B) Western blotting results for BACH1 in the placenta of WT and Bach1^-/-^ C57BL/6 mice treated with CA (*n* = 6). One-way analysis of variance followed by Tukey's post hoc analysis; ns: not significant, ***p* < 0.01, ****p* < 0.001.


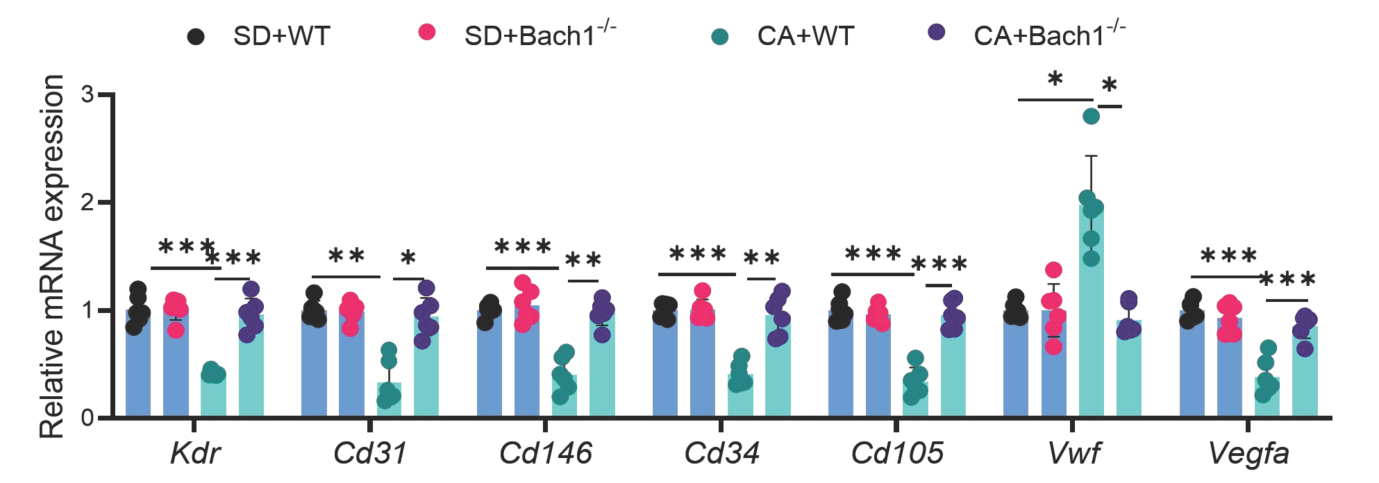


Fig. S4. BACH1 knockout improves CA-induced angiogenesis-related indicators in mice.

*Kdr, Cd31, Cd146, Cd34, Cd105, Vwf,* and *Vegfa* mRNA levels in placenta (*n* = 6). One-way analysis of variance followed by Tukey's post hoc analysis; ns: not significant, **p* < 0.05, ***p* < 0.01, ****p* < 0.001.


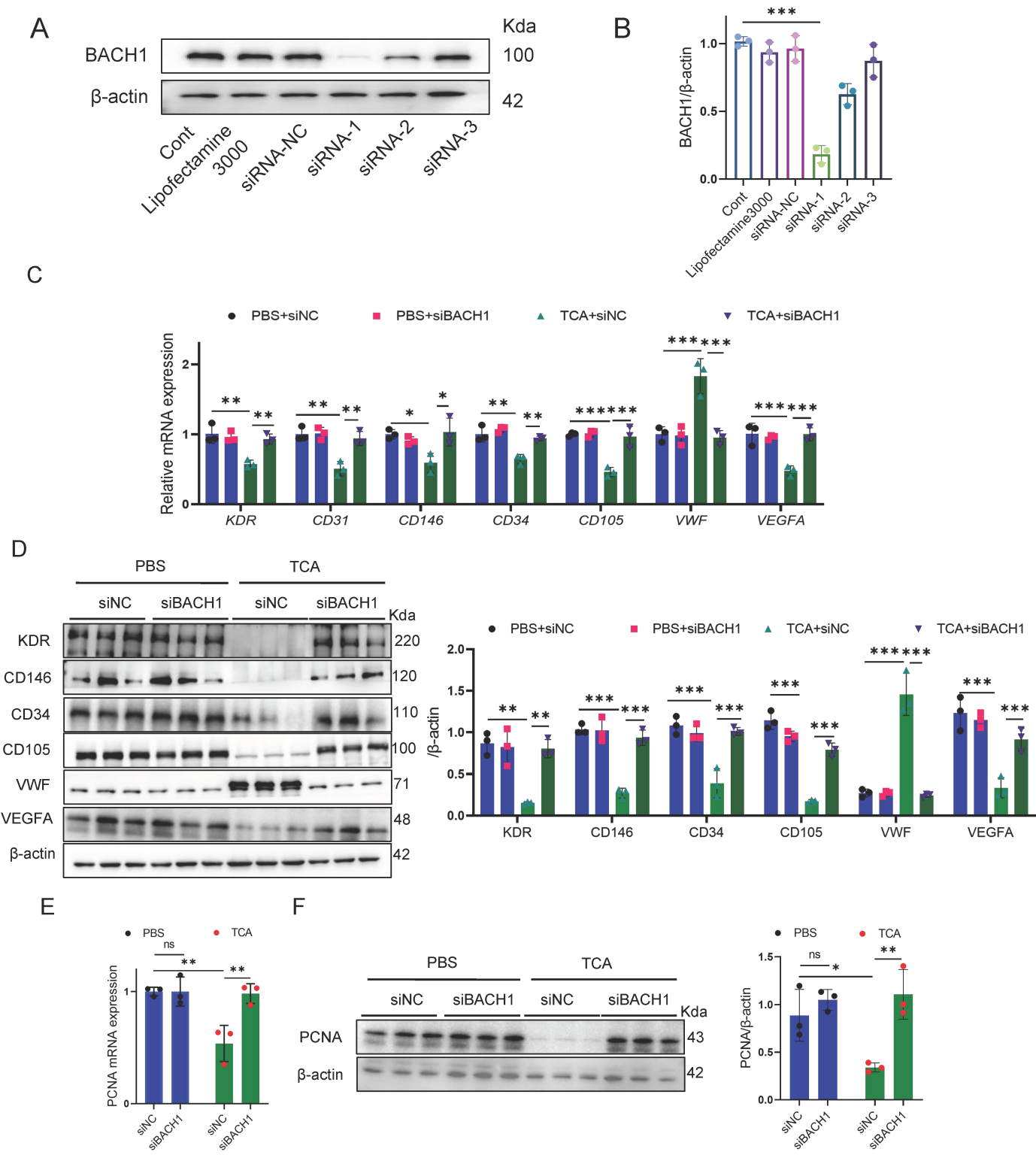


Fig. S5. BACH1 deficiency promotes taurocholic acid (TCA)-stimulated HUVEC proliferation.

(A, B) Western blotting for BACH1 in HUVECs (*n* = 3). (C) *KDR, CD31, CD146, CD34, CD105, VWF,* and *VEGFA* mRNA expression in siNC or siBACH1 HUVECs treated with TCA for 24 h (*n* = 3). (D) Western blotting of KDR, CD146, CD34, CD105, VWF, and VEGFA in siNC or siBACH1 HUVECs treated with TCA for 24 h (*n* = 3). (E) *PCNA* mRNA expression in siNC or siBACH1 HUVECs treated with TCA for 24 h (*n* = 3). (F) Western blotting of PCNA in siNC or siBACH1 HUVECs treated with TCA for 24 h (*n* = 3). *O*ne-way analysis of variance followed by Tukey's post hoc analysis; ns: not significant, **p* < 0.05, ***p* < 0.01, ****p* < 0.001.


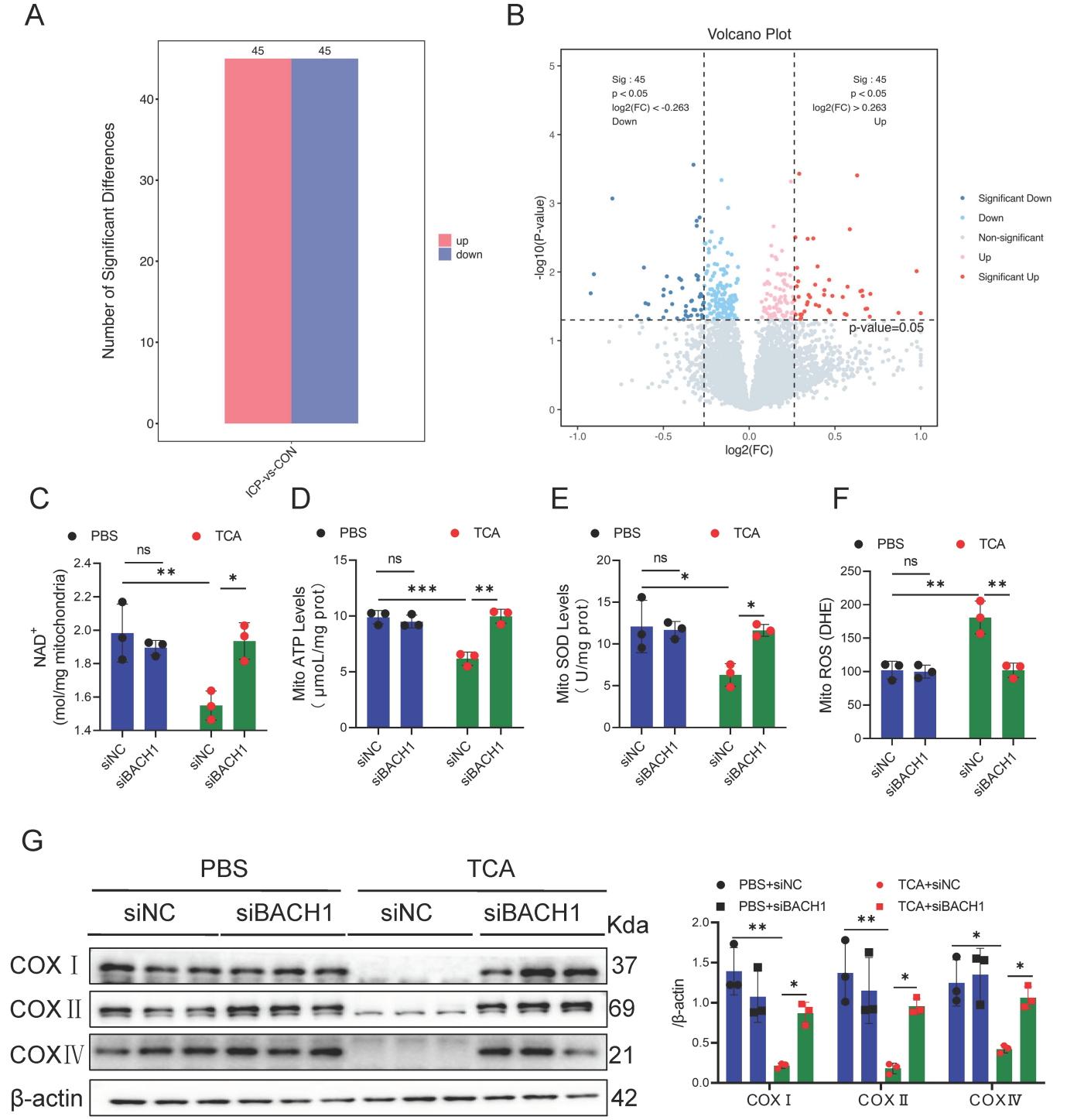


Fig. S6. BACH1 silencing promotes NAD+ transport to mitochondria.

(A) Analysis of differential protein expression. (B) Differential analysis of proteins in placental tissue; Blue: downregulated proteins, Red: upregulated proteins. (C–F) Quantification of NAD^+^, ATP, SOD, and ROS in mitochondria of siNC or siBACH1 HUVECs treated with taurocholic acid (TCA) for 24 h (*n* = 3). (G) Western blotting of COXI, COXII, and COXIV in siNC or siBACH1 HUVECs treated with TCA for 24 h (*n* = 3). One-way analysis of variance followed by Tukey's post hoc analysis; ns: not significant, **p* < 0.05, ***p* < 0.01, ****p* < 0.001.


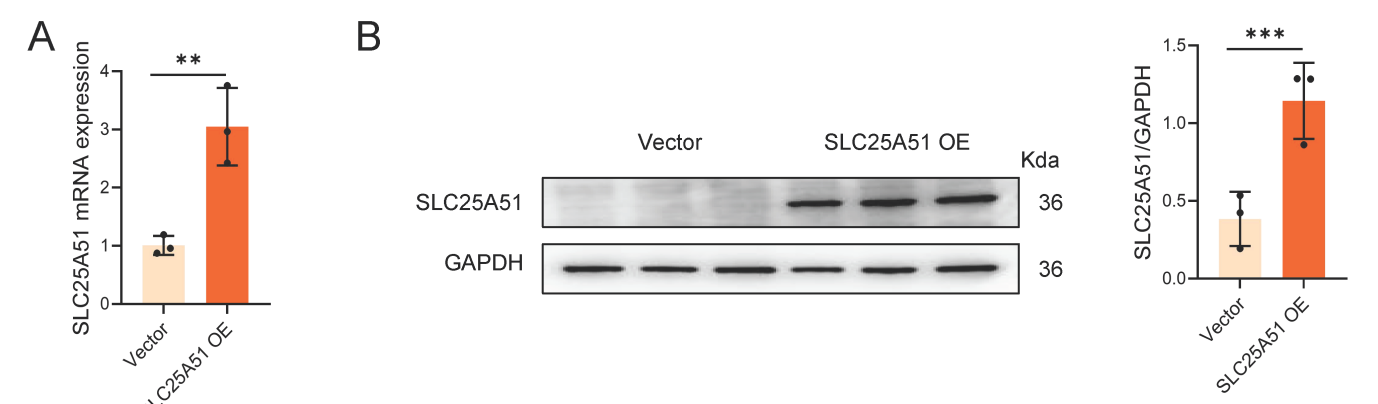


Fig. S7. Construction of SLC25A51-overexpressing (OE) cells.

(A) *SLC25A51* mRNA expression in vector or SLC25A51 OE HUVECs (*n* = 3). (B) Western blotting of SLC25A51 in vector or SLC25A51 OE HUVECs (*n* = 3). One-way analysis of variance followed by Tukey's post hoc analysis; ns: not significant, ***p* < 0.01, ****p* < 0.001.


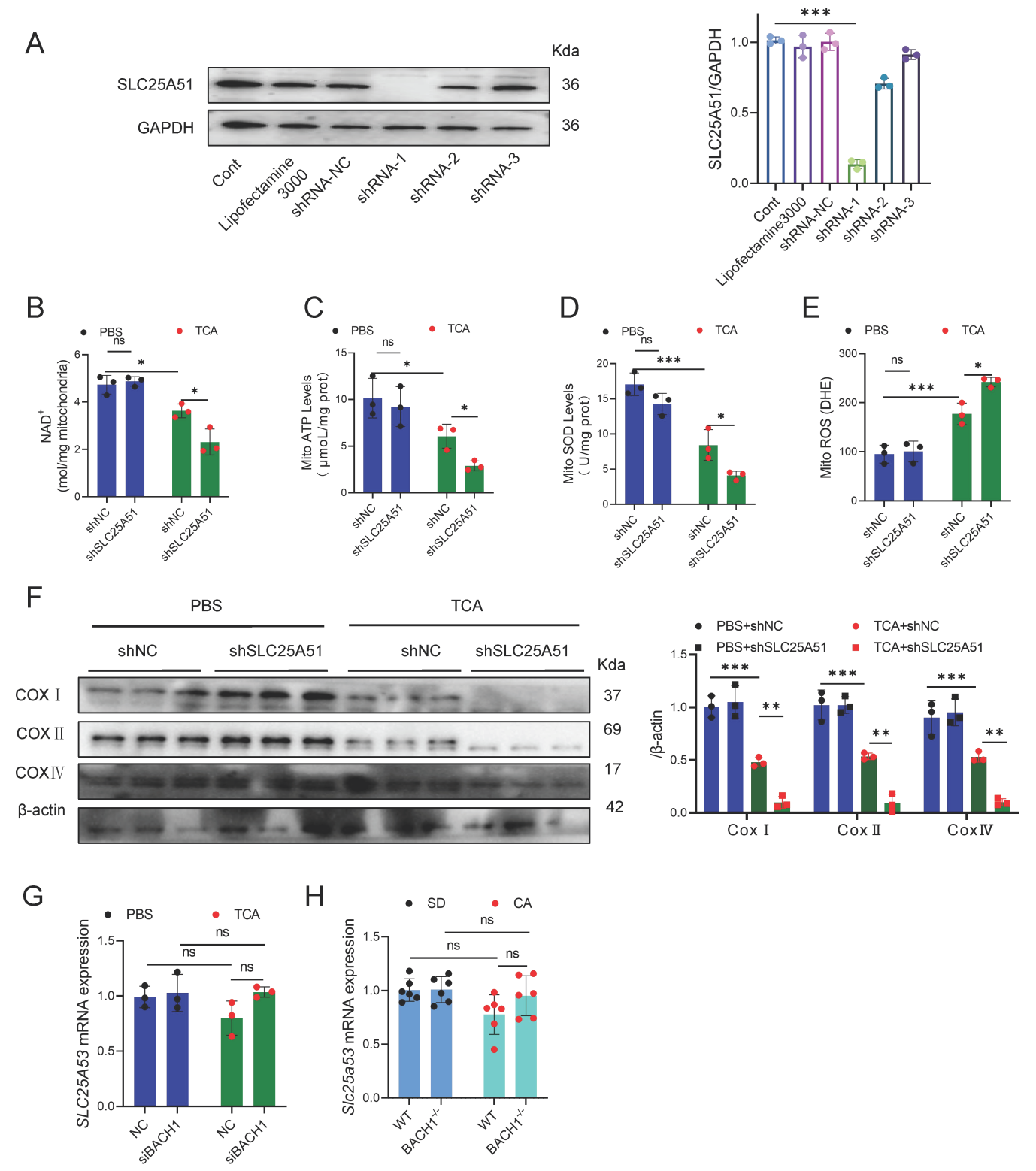


Fig. S8. SLC25A51 silencing aggravates mitochondrial damage.

(A) *SLC25A51* mRNA expression in shNC or shSLC25A51 HUVECs (*n* = 3). (B–E) Quantification of NAD+, ATP, SOD, and ROS in mitochondria of shNC or shSLC25A51 HUVECs treated with taurocholic acid (TCA) for 24 h (*n* = 3). (F) Western blotting of COXI, COXII, and COXIV in shNC or shSLC25A51 HUVECs treated with TCA for 24 h (*n* = 3). (G) *SLC25A53* mRNA expression in siNC or siBACH1 HUVECs treated with TCA for 24 h (*n* = 3). (H) *Slc25a53* mRNA expression in placenta of wild-type (WT) or BACH1^-/-^ C57BL/6 mice treated with CA (*n* = 6). Unpaired, two-tailed Student’s *t*-test or one-way analysis of variance followed by Tukey's post hoc analysis; ns: not significant, **p* < 0.05, ***p* < 0.01, ****p* < 0.001.


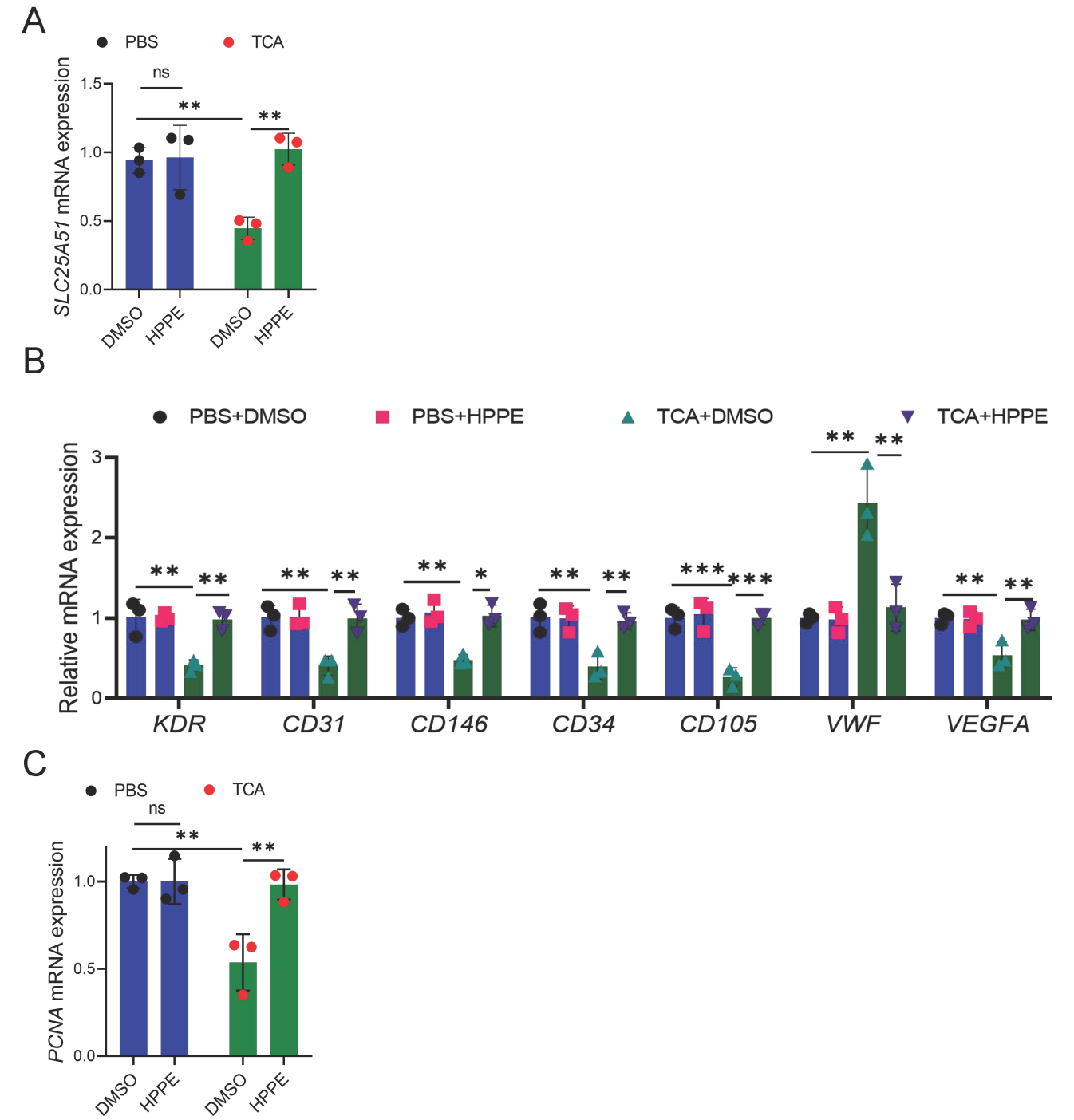


Fig. S9. BAHC1 inhibitor HPPE promotes taurocholic acid (TCA)-induced HUVEC proliferation.

(A) *SLC25A51* mRNA in HUVECs treated with HPPE (5 μM; *n* = 3). (B) *KDR, CD31, CD146, CD34, CD105, VWF,* and *VEGFA* mRNA expression in HUVECs treated with HPPE (5 μM; *n* = 3). (C) *PCNA* mRNA expression in HUVECs treated with HPPE (5 μM; *n* = 3). One-way analysis of variance followed by Tukey's post hoc analysis; ns: not significant, **p* < 0.05, ***p* < 0.01, ****p* < 0.001.
